# Trade-off between Responsiveness and Noise Suppression in Biomolecular System Responses to Environmental Cues

**DOI:** 10.1371/journal.pcbi.1002091

**Published:** 2011-06-30

**Authors:** Alexander V. Ratushny, Ilya Shmulevich, John D. Aitchison

**Affiliations:** Institute for Systems Biology, Seattle, Washington, United States of America; University of Chicago, United States of America

## Abstract

When living systems detect changes in their external environment their response must be measured to balance the need to react appropriately with the need to remain stable, ignoring insignificant signals. Because this is a fundamental challenge of all biological systems that execute programs in response to stimuli, we developed a generalized time-frequency analysis (TFA) framework to systematically explore the dynamical properties of biomolecular networks. Using TFA, we focused on two well-characterized yeast gene regulatory networks responsive to carbon-source shifts and a mammalian innate immune regulatory network responsive to lipopolysaccharides (LPS). The networks are comprised of two different basic architectures. Dual positive and negative feedback loops make up the yeast galactose network; whereas overlapping positive and negative feed-forward loops are common to the yeast fatty-acid response network and the LPS-induced network of macrophages. TFA revealed remarkably distinct network behaviors in terms of trade-offs in responsiveness and noise suppression that are appropriately tuned to each biological response. The wild type galactose network was found to be highly responsive while the oleate network has greater noise suppression ability. The LPS network appeared more balanced, exhibiting less bias toward noise suppression or responsiveness. Exploration of the network parameter space exposed dramatic differences in system behaviors for each network. These studies highlight fundamental structural and dynamical principles that underlie each network, reveal constrained parameters of positive and negative feedback and feed-forward strengths that tune the networks appropriately for their respective biological roles, and demonstrate the general utility of the TFA approach for systems and synthetic biology.

## Author Summary

Biological systems constantly balance noise suppression with responsiveness. In a fluctuating environment, some changes are insignificant to living cells while others represent cues to which they must respond. These stimuli are interpreted by molecular circuits that enable the cell to strike an appropriate balance between responsiveness and noise suppression. This trade-off is governed by the structure and kinetic parameters of molecular networks, which have been tuned by evolutionary selection for different stimuli and responses. We consider three regulatory circuits (two from yeast and one from mammalian cells), which respond to different environments and involve very different physiological processes. To investigate the responses to a time varying signal, we developed a generalized time-frequency analysis framework for studying such trade-offs using mathematical models of regulatory circuits and explore how the structure and parameters of the circuit affect the trade-offs between noise suppression and responsiveness. The generalized TFA approach represents an effective tool for exploring and analyzing different systems-level dynamical properties. Making use of such properties can facilitate prediction and network control for systems- and synthetic biology applications.

## Introduction

The living cell may be viewed as an information processing system that uses the information in its environment to make decisions and mount appropriate responses [1]–[3]. In this context, cellular systems must strike a balance between being highly responsive to the environment, preserving the necessary details of the signals that they process, while simultaneously exhibiting stability so as to suppress environmental noise that would otherwise confound the cell [4]. Indeed, the trade-off between noise suppression and detail preservation is a fundamental one even in engineered signal processing systems. An optimal system must extract the information from a signal in the presence of noise in order to make the most reliable estimate of some quantity of interest. Similarly, the cell must estimate the state of the extracellular environment from noisy input stimuli. For example, *E. coli* estimate the time derivative of a signal along which they chemotax [5]. This estimation is realized by a chemotactic network that essentially implements the Kalman filter [6], which optimally estimates the internal state of a linear dynamical system from a series of noisy measurements [7].

To discover evolutionarily conserved principles underlying cellular decision making, it is necessary to develop a general understanding of how biomolecular networks implement such trade-offs in terms of information processing, rather than in terms of specific biochemical details [4]. Mathematical models of molecular networks make it possible to quantitatively express these trade-offs in terms of input-output characteristics of the network and, in turn, to examine the effects of network topology (i.e., wiring) and parameters governing the interactions within the system.

The ability of a system to filter out fluctuations or to respond to temporal details in a signal can be captured quantitatively by analyzing the input and output signals in terms of their time and frequency characteristics. For instance, a system that performs smoothing of an input signal acts as a low-pass filter by attenuating high-frequency fluctuations. While frequency selective behavior of linear time-invariant systems is well understood and can be determined completely by knowing the response of the system to a single impulse [8], the nonlinear character of biological systems and the highly nonstationary nature of the input stimuli make it generally impossible to decouple system responses from their input signals. Indeed, the ability to quantitatively describe a response of a nonlinear biomolecular network to a transient stimulus, particularly in the context of varying strengths of combinatorial and synergistic regulatory interactions, typified by biological networks, remains a significant challenge.

Therefore, to explore how different network topologies confer particular system properties such as responsiveness and noise suppression, we developed a generalized time-frequency analysis (TFA) framework for investigating the responses of nonlinear biomolecular networks. The approach entails systematically comparing time-frequency characteristics of inputs (stimuli) and outputs (responses) while varying system parameters. This methodology allows for the exploration and quantitative comparisons of system-level behaviors relative to these parameters.

## Results/Discussion

### Time-frequency analysis of yeast metabolic responses to environmental change

**Figure 1:**
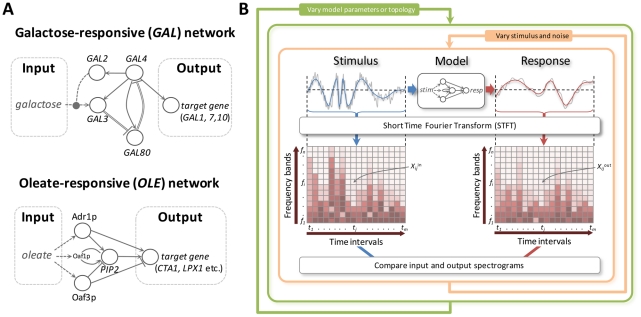
The generalized time-frequency analysis of the dynamical properties of molecular networks. (A) Schematic representations of the *GAL* (left) and *OLE* (right) networks. The *GAL* network is comprised of dual positive and negative feedback loops in which galactose activates Gal3p relieving Gal80p repression of Gal4p activity which upregulates the expression of *GAL* genes [11], [12]. The *OLE* network is comprised of overlapping positive and negative (coherent type 1 and type 2) feed-forward loops [24] in which oleate directly and indirectly activates core transcription factors (Oaf1p, Pip2p, Adr1p and Oaf3p) which regulate combinatorially target genes, such as the transcription factor *PIP2*, the catalase *CTA1*, the peroxisomal lipase *LPX1* and others [9], [10]. Networks are displayed as interactions of genes and gene products, which are not explicitly distinguished in the illustration. Solid lines terminating in arrowheads denote positive regulation whereas lines terminating in bars denote repression. Single solid lines represent protein-DNA interactions whereas double lines denote protein-protein interactions. Dotted arrows represent activation by the metabolite. The dotted oval arrow denotes activation of galactose transport. (B) Workflow of the generalized TFA. Noisy, time varying stimuli are used as inputs to a model of the network and the response is determined. Input and output signals are transformed into component frequencies by the STFT. The contribution of each frequency band (*f_i_*) at time interval (*t_j_*) is a spectrogram coefficient (*X_ij_*) and is represented in the spectrogram by a color intensity. Stimuli are varied randomly in the context of systematically varying model topologies and parameters.

We initially examined two yeast systems, the oleate (*OLE*) [9], [10] and the galactose (*GAL*) [11], [12] core transcriptional networks in *Saccharomyces cerevisiae*, which both respond to carbon source switching, but do so with very different network topologies (Figure 1A). The analysis of the spectral response of these systems to time varying inputs of simulated oleate and galactose concentrations makes it possible to study how the circuit structure and parameters in these two systems affect their ability to balance noise suppression with responsiveness. To this end, the time-frequency characteristics of the network input and output signals were extracted from their spectrograms, which are calculated using the short-time Fourier transform (STFT) [13]. The spectrogram illustrates how the frequency content of a signal varies with time. The value *X_i,j_* of each element in the spectrogram indicates the power of the signal at a particular frequency (*f_i_*) and at a particular time (*t_j_*) (Figures 1B and S1 and Text S1).

Characteristics of the signal and the network response can be quantified by integration across all frequency bands. Two characteristics of the networks, *noise suppression* (low-pass filtering) and *responsiveness* (detail preservation), can be inferred from the spectrograms. First, as a measure of circuit noise suppression, the spectrogram coefficients for each frequency band were summed over time and the mean frequency of the signal (μ) was calculated. The noise suppression characteristic (ξ) is defined as a ratio of mean frequencies of the stimulus and the system response (ξ = μ^in^/μ^out^, Figure S1). A greater ξ corresponds to a system with a greater ability to filter high-frequency input fluctuations. Second, in each frequency band, the relationship between the total variation of the signal power at the output and input of the system serves as a measure of responsiveness. Specifically, the total variation of the spectrogram coefficients within each frequency band (*V_i_*, Figure S1I) was calculated and the system responsiveness (ρ) is defined as the inverse divergence between distributions of normalized input (*V_i_* ^in*^) and output (*V_i_* ^out*^) variations across all frequency bands. A greater ρ corresponds to a more responsive system.

**Figure 2:**
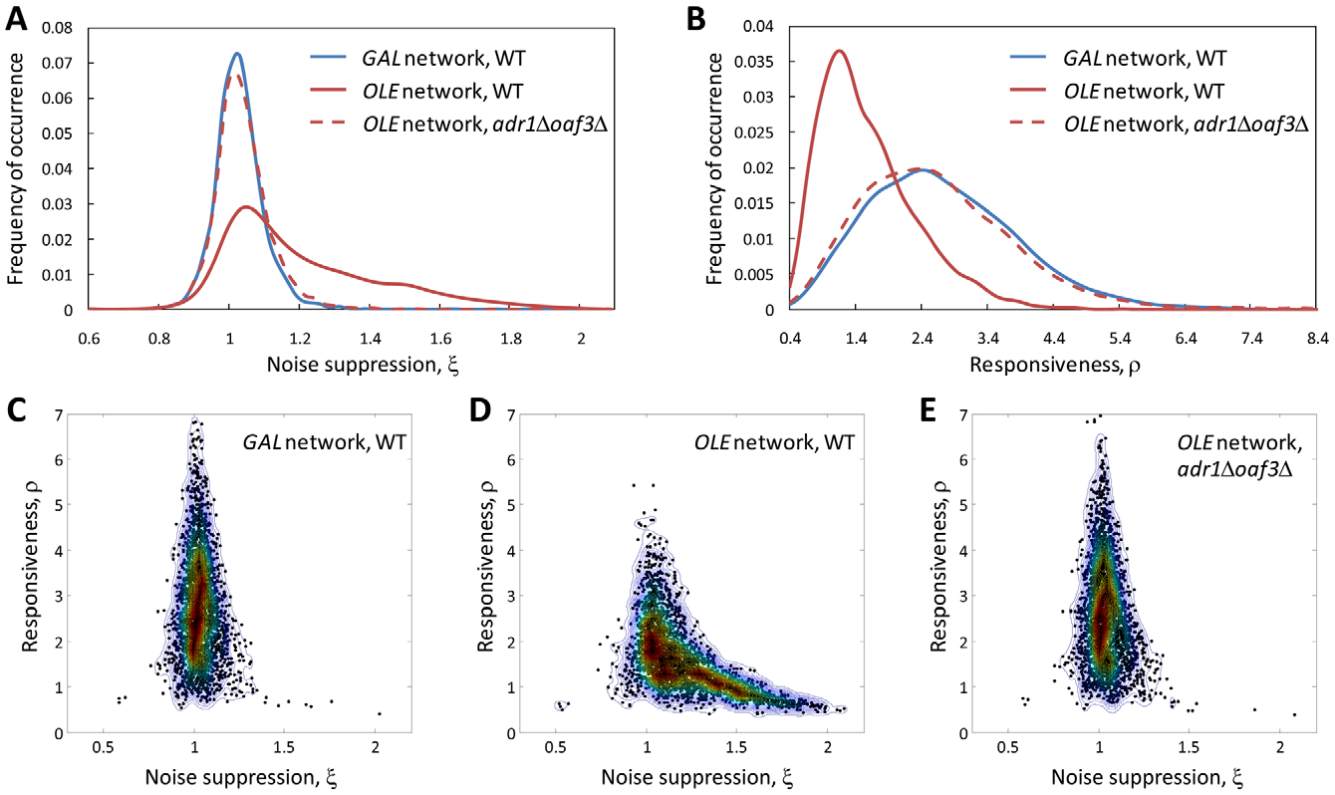
Feed-forward loops of the *OLE* network endow the system with ability to filter high-frequency fluctuations of the stimulus whereas feedback loops of the *GAL* network confer responsiveness to the environmental changes. (A, B) Distribution of the noise suppression and responsiveness statistics for *GAL* (WT) and *OLE* (WT or *adr1*Δ*oaf3*Δ) networks, respectively. (C–E) Responsiveness/noise suppression plots for *GAL* (WT) and *OLE* (WT, *adr1*Δ*oaf3*Δ) networks, respectively. The ξ and ρ were calculated based on 3000 random time-varying stimuli and system responses (see also Figures S3, S4, S5, S6 and S7). The contour plots were constructed using a bivariate Gaussian kernel density estimator (see Text S1). Noise suppression values less than 1 indicate the system responses to these stimuli contain higher harmonics than the input signals and *vice versa*. Low responsiveness values indicate poorly matched input and output signals. Δ denotes lack of a corresponding gene.

To investigate the different features of the *GAL* and *OLE* networks, their noise suppression (ξ) and responsiveness (ρ) characteristics were calculated based on their model-predicted responses to simulated random, noisy time-varying stimuli. The TFA analysis revealed that the two networks have distinct noise suppression and responsiveness properties. The *OLE* network effectively filters high-frequency fluctuations of the stimulus, thus acting as a low-pass filter. At the same time, it is relatively unresponsive to transient stimulus variations. By comparison, the *GAL* network is highly responsive but does not filter high frequency fluctuations as effectively (Figure 2). Thus, each system exhibits a different noise suppression-responsiveness trade-off, suggesting that these properties have selective advantages in different contexts.

The shift from glucose to oleate involves a substantial commitment to build and maintain new organelles (peroxisomes) that are responsible for metabolizing the new carbon source (fatty acids) [14], [15], a switch from fermentative to non-fermentative metabolism (requiring mitochondrial respiration), as well as the coordination of additional responses to the stress associated with exposure to fatty acids [9], [16]–[18]. Therefore, the nature of the oleate response demands that the system be capable of filtering high frequency fluctuations of the environmental stimulus, which may otherwise inappropriately commit the cell to significant morphological and metabolic reorganization. By contrast, the switch from glucose to galactose requires relatively few enzymes and transporters to convert galactose into glucose-1P for glycolysis [19]–[21]. Thus, while the ability of the cell to be highly responsive to galactose appears to come at the expense of noise suppression, such noise suppression can be sacrificed to a greater extent than during the oleate response.

A major difference between these networks lies in their topologies. The *GAL* network is comprised of dual positive and negative feedback loops (FBLs) whereas the *OLE* network is comprised of a positive FBL and two (positive and negative) feed-forward loops (FFLs) (Figure 1A). By removing coherent positive and negative FFLs (Adr1p and Oaf3p nodes) and leaving only the positive FBL (on *PIP2*), the ensemble of the calculated TFA statistics resembles that of the *GAL* network (Figure 2). To investigate how topology of the *OLE* network contributes to noise suppression and responsiveness of the system, different configurations of the *OLE* network were explored. Density distributions of the ξ and ρ for *adr1*Δ-, *oaf3*Δ-, “no positive feedback”-, “no positive feedback”-*adr1*Δ- and “no positive feedback”-*oaf3*Δ- *OLE* networks were calculated (Figures S4, S6 and S7 and Table S4). The “no positive feedback”-*OLE* model represents the *OLE* network where Pip2p does not upregulate its own gene *PIP2* but upregulates only its target genes.

The distributions of the TFA characteristics for the *adr1*Δ- and *oaf3*Δ-*OLE* models reveal that both Adr1p and Oaf3p individually increase the noise suppression and decrease the responsiveness of the *OLE* network (Figures S6D, S6E, S7D and S7E and Table S4). Interestingly, the *oaf3*Δ-*OLE* model has an even more narrowed noise suppression distribution with a lower mean ξ value than the *adr1*Δ*oaf3*Δ-*OLE* model (Table S4). The distributions of the ξ and ρ for the “no positive feedback”-*OLE* show that the Pip2p positive feedback decreases noise suppression and increases responsiveness of the *OLE* network (Figures S6F and S7F and Table S4). The responsiveness/noise suppression “TFA clouds” for the “no positive feedback”-*adr1*Δ-*OLE* and “no positive feedback”-*oaf3*Δ-*OLE* models (Figures S4G and H) are similar to the “TFA clouds” for the *adr1*Δ- and *oaf3*Δ-*OLE* models. This demonstrates that Adr1p and Oaf3p have more dominant contributions to the noise suppression and responsiveness characteristics than the Pip2p positive feedback. Overall these results reflect the nonlinear relationships between regulators in this regulatory network and suggest that the positive and negative FFLs of the *OLE* network serve to filter high frequency environmental fluctuations.

To examine how the noise suppression and responsiveness TFA characteristics depend on the type of random time-varying stimuli, the distributions of the ξ and ρ were calculated separately for each of the stimulus types (Figures S5, S6, and S7 and Table S4). The distribution of the noise suppression characteristic for the random sinusoidal stimuli is shifted toward higher values of ξ compared to the random “block” and “saw” stimuli regardless of the network type. This suggests that all of the biomolecular systems investigated here have a greater ability to suppress the noise of smoothed (random “sinusoidal”) rather than more abrupt (random “block” or “saw”) stimuli. The distribution of the responsiveness characteristic for the random sinusoidal stimuli is shifted toward lower values of ρ for the WT-, “no positive feedback”-*OLE* models and higher for WT-*GAL* and *oaf3*Δ-, “no positive feedback”-*oaf3*Δ- and *adr1*Δ*oaf3*Δ-*OLE* models compared to the random “block” and “saw” signals. The results indicate that the time-frequency characteristics of a biomolecular network does indeed depend on the nature of the stimulus, further supporting the approach of exploring the network responses to a large ensemble of random time-varying stimuli.

**Figure 3:**
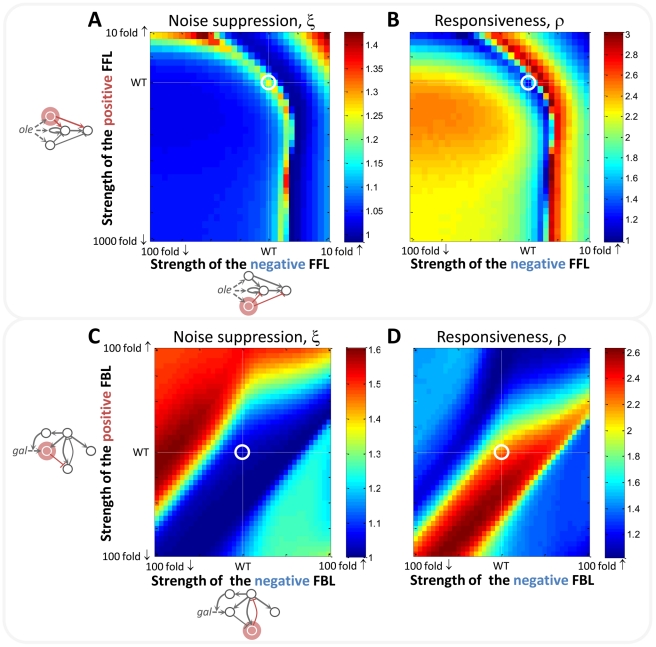
The noise suppression (ξ) and the responsiveness (ρ) of the networks are sensitive to both to the topologies and rate parameters. (A, B) The ξ and ρ, respectively, of the *OLE* network as a function of the strengths of positive and negative FFLs. (C, D) The ξ and ρ, respectively, of the *GAL* network as a function of the strengths of positive and negative FBLs. Each point on the heat maps represents the averaged ξ and ρ, respectively, over 100 random and noisy stimuli (see also Figures S11 and S12). The strengths of the FFLs/FBLs are on a logarithmic scale. White lines represent WT parameters and their encircled intersection is the WT network.

To understand the responsiveness and noise suppression properties of networks, typified by the interlinked positive and negative FBLs of the *GAL* network and the interlinked negative and positive FFLs of the *OLE* network, the parameters corresponding to the strengths of the FFLs and FBLs were systematically altered. The strength of each loop was independently varied (2,642 parameter sets in total) and each parameter set was explored with 100 randomized model inputs. The noise suppression and responsiveness characteristics of the networks as a function of network parameters were determined by TFA and displayed as heat maps (Figure 3). The resulting “portraits” expose fundamental differences inherent to each of the networks and demonstrate how network dynamics can be predicted for these and evolutionarily conserved networks.

For the *OLE* network, TFA revealed that network behavior is characterized by appropriately tuned opposing positive and negative FFLs. The network is maximally stable along the arc-like front shown in Figure 3A. Increasing the strengths of the positive and/or negative FFLs above this front results in non-physiological responses, characterized by a reversed directionality of the output relative to the input signals (Figure S8). Decreasing the strengths of these FFLs below the front results in lowered noise suppression at the expense of increased system responsiveness (Figure 3). As might be expected for a nonlinear system, an increase in noise suppression may not be reflected by a corresponding decrease in responsiveness of the same magnitude and vice versa. While the arc-shaped front represents the range of parameters where the noise suppression and responsiveness characteristics are similar to the wild-type (WT) state (Figures S10A and S10B and Table S5), there are other trade-offs as parameters vary along this arc; for example, the amplitude of the response changes as a function of the strengths of the positive and negative FFLs (Figure S9).

By contrast, the dual negative and positive FBL in the *GAL* network is highly responsive over a broad range of parameters extending along the diagonal from the left bottom corner of the heat map (Figure 3D). The strengths of positive and negative FBLs can be varied over an extensive parameter range, while maintaining near WT responsiveness and noise suppression, indicating a remarkable level of robustness of the system (Figures S10C and S10D and Table S5). Indeed, while the *GAL* network does have the capacity to act as a low-pass filter [1], a significant deviation from WT parameters would be required for this system to reach the effectiveness of the *OLE* network in terms of low-pass filtering (Figure 3C). Based on these simulations, decreasing the strength of the negative FBL with the fixed strength of the positive FBL, would increase the noise suppression of the network and decrease its responsiveness (Figures 3C and 3D).

To investigate how the TFA portraits depend on the type of random time-varying stimuli, the heat maps presented in Figure 3 were split into three separate heat maps, each of which represents an averaged ξ/ρ over 33/34/33 random “block”/sinusoidal/“saw” stimuli. The separated heat maps for the *OLE* (Figure S11) and the *GAL* (Figure S12) models show similar patterns within each model for different types of stimuli; however, the ranges of ξ and ρ values (as the strengths of FFLs and FBLs are changed) differ depending on type of stimulus. For example, the difference between maximum and minimum ξ and ρ values of the “sinusoidal” heat maps is greater compared to the “block” or “saw” stimuli regardless of the network. This analysis highlights that TFA portraits (in this case, the projection onto the plane of positive and negative FFL/FBL strengths) tend to be robust to changes in stimulus type in terms of the patterns of ξ and ρ changes in the parameter space (as shown in Figures S11 and S12).

### The LPS-induced regulatory network in macrophages

**Figure 4:**
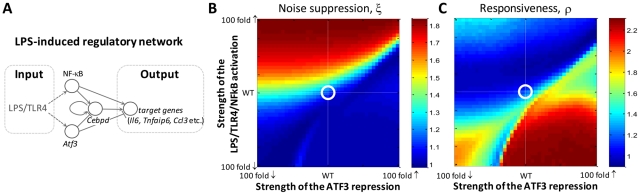
LPS-induced regulatory network driven by NF-κB, ATF3 and C/EBPδ transcription factors lies at the boundary of responsiveness and noise suppression. (A) Schematic representation of the LPS-induced regulatory network. The network is comprised of overlapping positive and negative (coherent type 1 and type 2) feed-forward loops [24] in which LPS indirectly activates core transcription factors (NF-κB and ATF3) which regulate combinatorially target genes, such as the transcription factor C/EBPδ, interleukin 6 (IL6), tumor necrosis factor, alpha-induced protein 6 (TNFAIP6), Ccl3 chemokine and others [22]. Network notation is described in Figure 1. (B, C) The ξ and ρ, respectively, of the LPS-induced regulatory network as a function of the strengths of the LPS/TLR4/NFkB activation and the ATF3 repression (see Text S1). Each point on the heat maps represents the averaged ξ and ρ, respectively, over 100 random and noisy stimuli (see also Figure S13). The strengths of the LPS/TLR4/NFkB activation and the ATF3 repression are on a logarithmic scale. White lines represent WT parameters and their encircled intersection is the WT network.

To investigate the extent to which overall network architecture (versus biochemical parameters) defines the dynamical properties of a system, we examined the LPS and Toll-like receptor 4 (TLR4)-induced regulatory circuit from mouse macrophages by TFA, which, like the *OLE* network, is characterized by overlapping positive and negative coherent FFLs. In this regulatory network NF-κB, ATF3 and C/EBPδ transcription factors coordinate the expression of cytokine encoding target genes in response to LPS [22] (Figure 4A). The network is also interesting because it must be tightly regulated to respond vigorously to the presence of a pathogen, but at the same time must remain in check to avoid uncontrolled inflammatory responses. Analogously to the *OLE* and *GAL* models, the behavior of the LPS-induced network model was initially investigated in the wild-type state in the presence of 3000 random and noisy stimuli (Figures S4C, S6C and S7C and Table S4). The WT-LPS model has a similar responsiveness/noise suppression distribution as the *OLE* model, i.e. biased toward more noise suppression. The distribution of the noise suppression and responsiveness TFA characteristics for different stimulus types are presented in Figures S5C, S6C and S7C and Table S4.

Similarly to the *OLE* model, the strength of each FFL of the LPS-induced network was independently varied and each parameter set was explored with 100 randomized model inputs. The heat maps of the TFA characteristics resulting from this simulation were significantly different from those of the *OLE* network emphasizing that the network architecture itself is not sufficient to define the dynamical characteristics of the system. Indeed, while the *OLE* network appears tuned toward greater noise suppression, the LPS network appears to be tuned to lie at the boundary of responsiveness and noise suppression (Figures 4B, 4C, S10E, S10F, S13). This is perhaps not surprising considering that macrophages must be finely tuned to respond to the presence of a foreign substance, yet if cellular responses vary dramatically with the character of the signal, variations in cytokine release has the potential to lead to inappropriate inflammatory responses.

The ability to be poised at the boundary of responsiveness and stability is a hallmark of systems operating in a critical regime between order and disorder. A recent study of mouse macrophages stimulated with a variety of pathogen associated molecular patterns provided evidence that macrophages gene expression dynamics are indeed critical [23], supporting the conclusions drawn from the TFA analysis.

Deviation from the parameters that define the wild-type network has a dramatic effect on the network behavior. Increasing the strength of LPS/TLR4/NFkB activation from the WT state increases the noise suppression of the network, but at the cost of reducing responsiveness. Similarly, decreasing the strength of the activating arm increases responsiveness, but at the cost of reducing noise suppression. Changing the strength of ATF3 repression leads to an opposite pattern with less dramatic changes in network behavior. Thus, altering the strength of either the positive or negative FFLs leads to networks that are predicted to change the finely tuned balance between noise suppression and responsiveness that is critical to a controlled inflammatory response.

### Conclusion

While experimental tools of systems biology allow us to discern molecular network structures, it is evident that the parameters governing the interactions within the system are essential for understanding its dynamics. However, in most cases, one has only partial knowledge of the parameter values in the system, with many parameters being either entirely undetermined or known only imprecisely. The generalized TFA framework is particularly useful in such scenarios as it can reveal various aspects of dynamical system behavior such as noise suppression, responsiveness, and their trade-offs, relative to the parameter space of the system. Moreover, other dynamical properties of a network can be investigated in the same manner by extracting appropriate features from the time-frequency representations or other metrics for features such as noise suppression and responsiveness can readily be incorporated and compared in the TFA framework. Additionally, the generalized TFA framework is not constrained by the STFT; wavelets or other multiresolution or multiscale analysis approaches can also be used for time-frequency representations. The noise suppression and responsiveness portraits of the *OLE*, the *GAL* and the LPS-induced networks (Figures 3, 4B and 4C) reveal radically different behaviors and biological roles for these circuits. Such portraits can also suggest new avenues for experimental research in synthetic biology aimed at modulating the biochemical properties of the interactions to affect systems-level trade-offs, while maintaining physiologically viable responses.

## Methods

### Computational modeling

To systematically explore the dynamical properties of the *OLE*, *GAL* and LPS-induced networks three basic types of random noisy stimuli (“block”, “saw”, and sinusoidal signals) were used. Random stimuli were generated using *precalcInputSignals* MATLAB function (http://magnet.systemsbiology.net/tfa). The amplitude range of the generated random time variant stimuli was scaled so that the maximum amplitude of the stimulus for the *GAL* network corresponds to 11.1 mM of external galactose, the maximum amplitude of the stimulus for the *OLE* network corresponds to 4.25×10^−6^ M of intracellular oleate and the maximum amplitude of the stimulus for the LPS-induced network corresponds to 1,500 molecules/cell of the nuclear NF-κB. The duration of the generated random time variant stimuli was scaled to be equal to 3000 min. The ordinary differential equation (ODE) kinetic models of the *OLE* [10] (see also Text S1 and Tables S1, S2 and S3) and *GAL* [12] (see also Text S1) networks and the delay differential equation (DDE) kinetic model of the LPS-induced [22] network were solved using the standard ODE and DDE solvers, respectively, in MATLAB. Each experiment in Figure 2 consists of 3000 stimuli (1000 random “block” signals, 1000 random “saw” signals, and 1000 random sinusoidal signals; see Figure S2). Heat maps in Figures 3, 4B and 4C were constructed based on 33 “block”, 33 “saw”, and 34 sinusoidal random signals. Model responses (the target gene expression profiles) to random noisy stimuli were calculated using MATLAB functions that are available from http://magnet.systemsbiology.net/tfa. More extensive details of these calculations are given in Text S1.

### Construction of spectrograms

Spectrograms were constructed using the *PlotSpectrogram* MATLAB routine (http://www.mathworks.com/matlabcentral/fileexchange). The spectrogram coefficients in the routine were calculated as 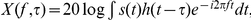 where 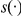 is the signal to be transformed and 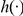 is the Hamming window function. Each spectrogram was preprocessed prior to subsequent analysis. Coefficients of each spectrogram less than 80 dB below the maximum were set to zero. Then each spectrogram coefficient (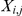) was scaled by a factor of 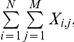 where *N* and *M* are the number of frequency bands and time intervals, respectively. The illustration of the time-frequency analysis of the network responses to the random noisy “block” stimulus is presented in Figure S1.

### Analysis of spectrograms

The mean frequency of the signal was calculated as 
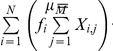
 The total variation of the spectrogram coefficients within each frequency band *f_i_* is defined as 
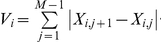
 The system responsiveness, ρ, was calculated as the inverse symmetric Kullback-Leibler divergence between the normalized distributions of *V* ^in*^ and *V* ^out*^ across all frequency bands 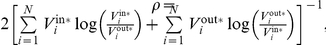 where 
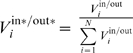
 Other statistics for the noise suppression (the Kullback-Leibler ansd Kolmogorov-Smirnov distances) and responsiveness (the inverse Kolmogorov-Smirnov distance) characteristics of the *OLE* and *GAL* networks were also calculated (Figure S3). These are consistent with the results presented in Figure 2. The noise suppression and responsiveness statistics were calculated using the *getHMforAllVarPrms* MATLAB function (http://magnet.systemsbiology.net/tfa).

Complete details of all methods used and the specifics of computational models are available in Text S1.

## Supporting Information

Figure S1 Illustration of the time-frequency analysis of the *OLE* and *GAL* network responses to the random noisy “block” stimulus. (A–C) Zero-mean stimulus and the *OLE* and *GAL* model responses, respectively. (D–F) Spectrograms of the zero-mean stimulus and the *OLE* and *GAL* model responses, respectively. (G–H) The spectrogram coefficient sum distributions across frequency bands and corresponding cumulative distributions, respectively. (I–J) The spectrogram coefficient total variation distributions across frequency bands and corresponding cumulative distributions, respectively.
(TIF)

Figure S2 Examples of the *OLE* and *GAL* network responses to random noisy stimuli. (A) The *OLE* and *GAL* network responses to random noisy “block” stimuli. (B) The *OLE* and *GAL* network responses to random noisy “saw” stimuli. (C) The *OLE* and *GAL* network responses to random noisy sinusoidal stimuli.
(TIF)

Figure S3 Responsiveness/noise suppression contour plots for (A, B, C) *GAL*, (D, E, F) WT-*OLE*, and (G, H, I) *adr1*Δ*oaf3*Δ-*OLE* networks. Contour plots were calculated based on TFA characteristics of 3000 random stimuli/system responses (see Figure 2 in the main text). ξ_1_ denotes the symmetric Kullback-Leibler divergence between the distributions of the input and output spectrogram coefficient sums across all frequency bands. ξ_2_ denotes the Kolmogorov-Smirnov distance between the distributions of the input and output spectrogram coefficient sums across all frequency bands. ρ_1_ denotes the inverse Kolmogorov-Smirnov distance between the normalized total variation distributions of the input and output spectrogram coefficients across all frequency bands. The noise suppression and responsiveness statistics were calculated using the *getHMforAllVarPrms* MATLAB function (http://magnet.systemsbiology.net/tfa).
(TIF)

Figure S4 Responsiveness/noise suppression plots for (A) WT-*GAL*, (B) WT-*OLE*, (C) WT-LPS, (D) *adr1*Δ-*OLE*, (E) *oaf3*Δ-*OLE*, (F) “no positive feedback”-*OLE*, (G) “no positive feedback”-*adr1*Δ-*OLE*, (H) “no positive feedback”-*oaf3*Δ-*OLE*, (I) *adr1*Δ*oaf3*Δ-*OLE* models. The ξ and ρ were calculated based on 3000 random time-varying stimuli and system responses. The contour plots were constructed using a bivariate Gaussian kernel density estimator (see Figure 2 in the main text and Figures S3, S5, S6 and S7). The “no positive feedback”-*OLE* model represents the *OLE* network where Pip2p does not upregulate its own gene *PIP2* but upregulates its target genes.
(TIF)

Figure S5 Colored scatter plots of the noise suppression and responsiveness statistics for (A) WT-*GAL*, (B) WT-*OLE*, (C) WT-LPS, (D) *adr1*Δ-*OLE*, (E) *oaf3*Δ-*OLE*, (F) “no positive feedback”-*OLE*, (G) “no positive feedback”-*adr1*Δ-*OLE*, (H) “no positive feedback”-*oaf3*Δ-*OLE*, (I) *adr1*Δ*oaf3*Δ-*OLE* models. The ξ and ρ were calculated based on 3000 random time-varying stimuli and system responses (see Figure 2 in the main text and Figures S3, S4, S6 and S7). The color of the dots represents the type of stimuli applied to the networks. The blue, red and green dots represent “block”, sinusoidal, and “saw” signals, respectively. The “no positive feedback”-*OLE* model represents the *OLE* network where Pip2p does not upregulate its own gene *PIP2* but upregulates its target genes.
(TIF)

Figure S6 Distribution of the noise suppression characteristic for (A) WT-*GAL*, (B) WT-*OLE*, (C) WT-LPS, (D) *adr1*Δ-*OLE*, (E) *oaf3*Δ-*OLE*, (F) “no positive feedback”-*OLE*, (G) “no positive feedback”-*adr1*Δ-*OLE*, (H) “no positive feedback”-*oaf3*Δ-*OLE*, (I) *adr1*Δ*oaf3*Δ-*OLE* models. The ξ was calculated based on 3000 random time-varying stimuli and system responses (see Figure 2 in the main text and Figures S3, S4, S5 and S7). The color of the density plots represents the type of stimuli applied to the networks. The blue, red, green and black density distributions represent random “block”, sinusoidal, “saw” and all together stimuli, respectively. The “no positive feedback”-*OLE* model represents the *OLE* network where Pip2p does not upregulate its own gene *PIP2* but upregulates its target genes.
(TIF)

Figure S7 Distribution of the responsiveness characteristic for (A) WT-*GAL*, (B) WT-*OLE*, (C) WT-LPS, (D) *adr1*Δ-*OLE*, (E) *oaf3*Δ-*OLE*, (F) “no positive feedback”-*OLE*, (G) “no positive feedback”-*adr1*Δ-*OLE*, (H) “no positive feedback”-*oaf3*Δ-*OLE*, (I) *adr1*Δ*oaf3*Δ-*OLE* models. The ρ was calculated based on 3000 random time-varying stimuli and system responses (see Figure 2 in the main text and Figures S3, S4, S5 and S6). The color of the density plots represents the type of stimuli applied to the networks. The blue, red, green and black density distributions represent random “block”, sinusoidal, “saw” and all together stimuli, respectively. The “no positive feedback”-*OLE* model represents the *OLE* network where Pip2p does not upregulate its own gene *PIP2* but upregulates its target genes.
(TIF)

Figure S8 Physiological vs. non-physiological network responses. (A, B) The Euclidian distance between input and output derivatives of the *OLE* and *GAL* networks as a function of the strengths of positive and negative FFLs and FBLs, respectively. Each point on the heat maps represents the averaged Euclidian distance over 100 random and noisy stimuli (see Figure 3 in the main text). The strengths of the FFLs/FBLs are on a logarithmic scale. Non-physiological range of parameters for the *OLE* model is surrounded by the gray curve. (C) Example of a non-physiological response of the *OLE* model, which corresponds to the encircled area on the heat map (A). (D) Example of a physiological response of the *GAL* model, which corresponds to the encircled area on the heat map (B). There are no obvious non-physiological responses for the *GAL* model in the explored parameter space.
(TIF)

Figure S9 Amplitude of (A) *OLE* and (B) *GAL* network responses as a function of positive and negative FFL and FBL strengths, respectively. Each point on the heat maps represents the averaged amplitude over 100 random and noisy stimuli (see Figure 3 in the main text). The strengths of the FFLs/FBLs are on a logarithmic scale. White lines represent WT parameters and their encircled intersection is the WT network.
(TIF)

Figure S10 Noise suppression and responsiveness heat map areas within ±15% of the wild type ξ and ρ values for (A, B) *OLE*, (C, D) *GAL* and (E, F) LPS models, respectively. Heat map areas with ξ and ρ values below or above the threshold (±15% of WT values) are set to be equal to the minimum or the maximum value of the heat map, respectively. White lines represent WT parameters and their encircled' intersection is the WT network.
(TIF)

Figure S11 The noise suppression (ξ) and the responsiveness (ρ) of the *OLE* model as a function of the positive and negative FFL strengths. Each point on the heat maps represents the averaged ξ or ρ over (A, B) 33 random “block” or (C, D) 34 random sinusoidal or (E, F) 33 random “saw” stimuli. The strengths of the FFLs are on a logarithmic scale. White lines represent WT parameters and their encircled intersection is the WT network.
(TIF)

Figure S12 The noise suppression (ξ) and the responsiveness (ρ) of the *GAL* model as a function of the positive and negative FBL strengths. Each point on the heat maps represents the averaged ξ and ρ over (A, B) 33 random “block” or (C, D) 34 random sinusoidal or (E, F) 33 random “saw” stimuli. The strengths of the FBLs are on a logarithmic scale. White lines represent WT parameters and their encircled intersection is the WT network.
(TIF)

Figure S13 The noise suppression (ξ) and the responsiveness (ρ) of the LPS model as a function of the positive and negative FFL strengths. Each point on the heat maps represents the averaged ξ and ρ over (A, B) 33 random “block” or (C, D) 34 random sinusoidal or (E, F) 33 random “saw” stimuli. The strengths of the FFLs are on a logarithmic scale. White lines represent WT parameters and their encircled intersection is the WT network.
(TIF)

Table S1 Equations defining the *OLE* network model.
(DOC)

Table S2 Dynamic variables of the *OLE* network model.
(DOC)

Table S3 Kinetic parameters and derived parameters used in the *OLE* network model.
(DOC)

Table S4 Mean, standard deviation (SD) and coefficient of variation (CV) values of the noise suppression (ξ) and responsiveness (ρ) characteristics for the WT-*GAL*, WT-*OLE*, WT-LPS, *adr1*Δ-*OLE*, *oaf3*Δ-*OLE*, “no positive feedback”-*OLE*, “no positive feedback”-*adr1*Δ-*OLE*, “no positive feedback”-*oaf3*Δ-*OLE* and *adr1*Δ*oaf3*Δ-*OLE* models calculated for 1000 “block”, 1000 “saw”, and 1000 sinusoidal random signals (see Figure 2 in the main text and Figures S3, S4, S5, S6 and S7).
(DOC)

Table S5 Mean values of the noise suppression (ξ) and responsiveness (ρ) WT characteristics for the *GAL*, *OLE* and LPS models calculated for 33 “block”, 33 “saw”, and 34 sinusoidal random signals (see Figures 3 and 4 in the main text).
(DOC)

Text S1 This document contains additional supplemental information on the generation of random stimuli, mathematical models and computational methods used in this work.
(DOC)
