# Supplementary figures and images for: Trade-off between Responsiveness and Noise Suppression in Biomolecular System Responses to Environmental Cues
Source: PLoS Comput Biol. 2011 Jun 30;7(6):e1002091. doi: 10.1371/journal.pcbi.1002091 (PMC3127798; doi:10.1371/journal.pcbi.1002091)

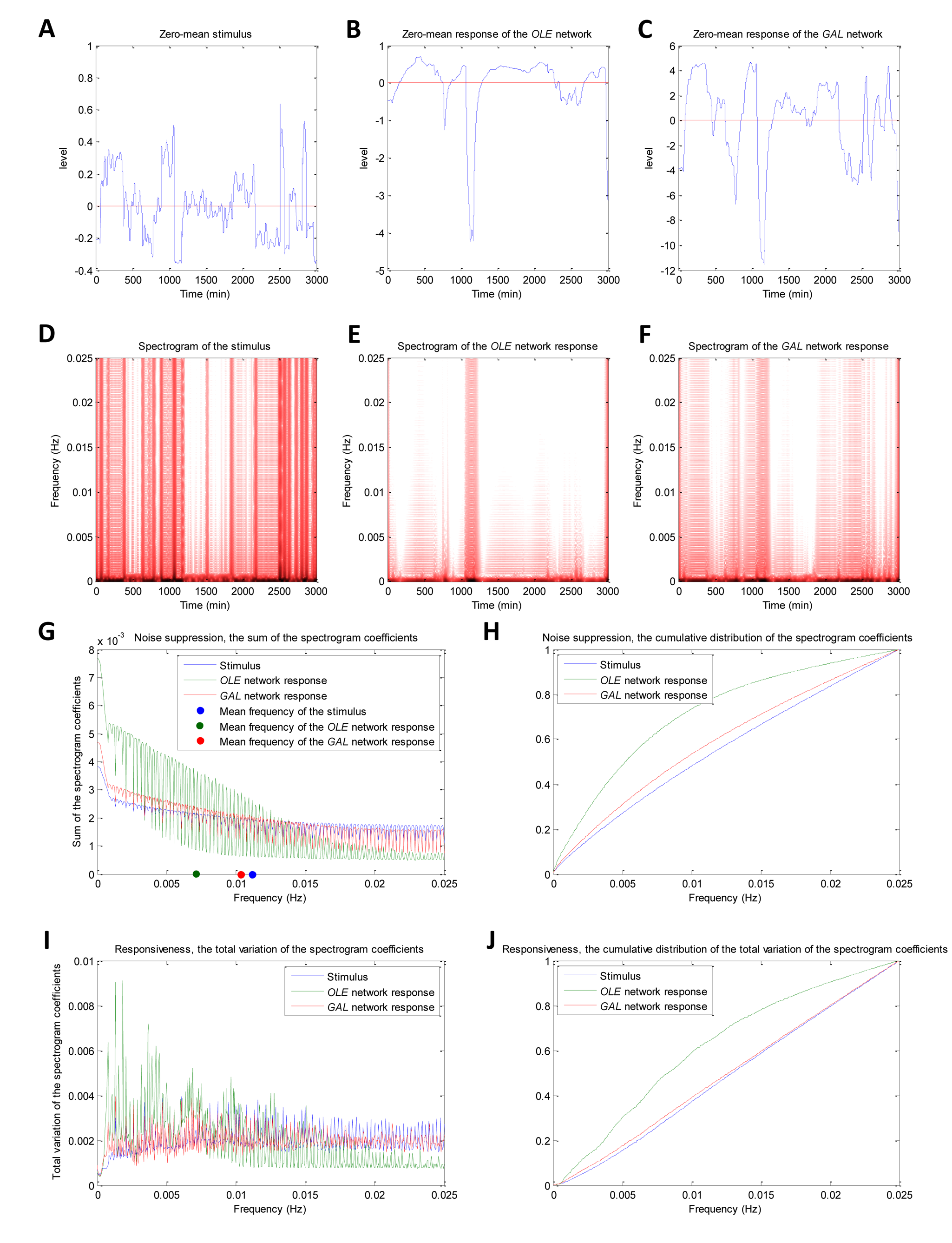

Supplement: Figure S1 — Illustration of the time-frequency analysis of the OLE and GAL network responses to the random noisy “block” stimulus. (A–C) Zero-mean stimulus and the OLE and GAL model responses, respectively. (D–F) Spectrograms of the zero-mean stimulus and the OLE and GAL model responses, respectively. (G–H) The spectrogram coefficient sum distributions across frequency bands and corresponding cumulative distributions, respectively. (I–J) The spectrogram coefficient total variation distributions across frequency bands and corresponding cumulative distributions, respectively. (TIF) [file pcbi.1002091.s001.tif]

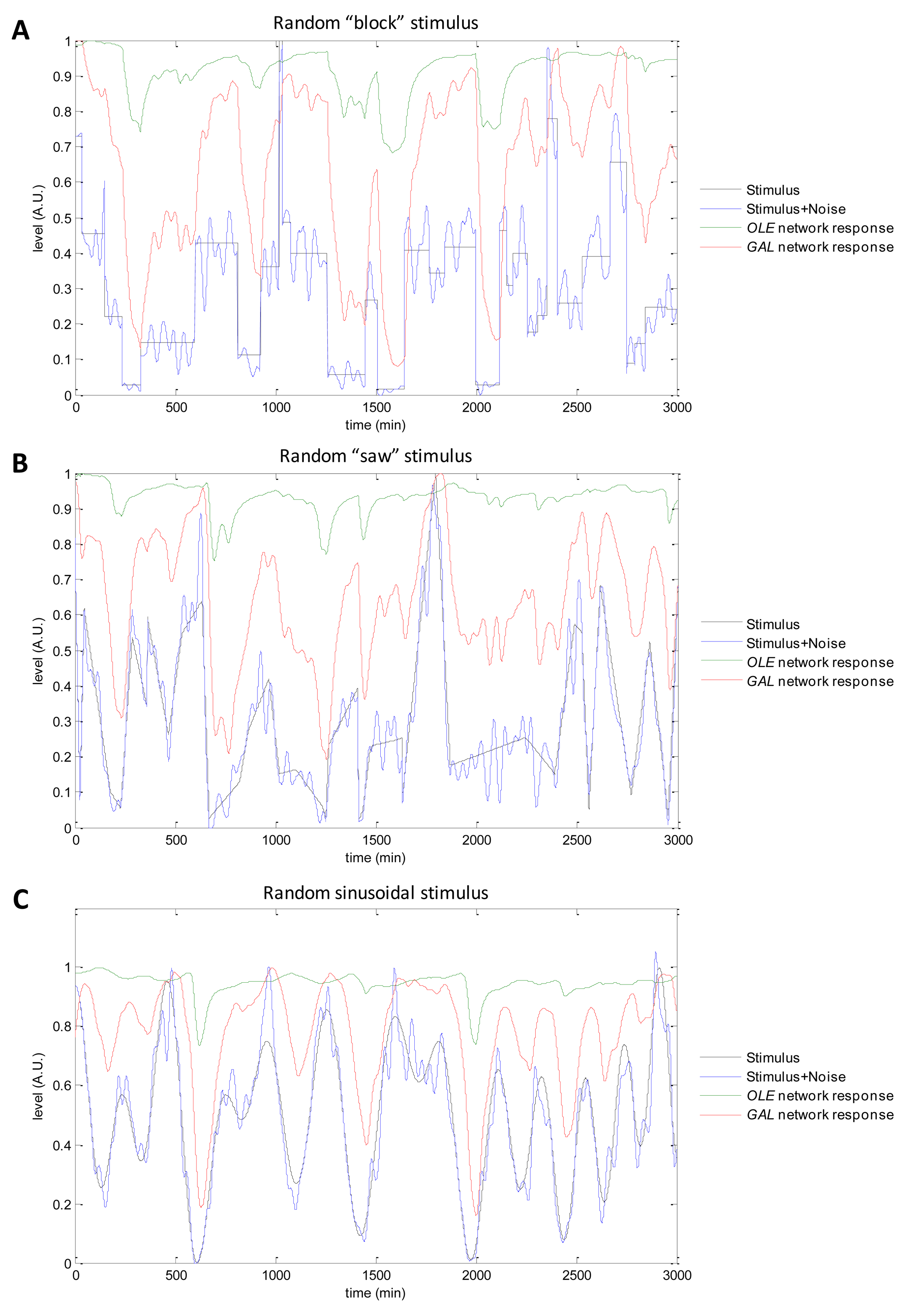

Supplement: Figure S2 — Examples of the OLE and GAL network responses to random noisy stimuli. (A) The OLE and GAL network responses to random noisy “block” stimuli. (B) The OLE and GAL network responses to random noisy “saw” stimuli. (C) The OLE and GAL network responses to random noisy sinusoidal stimuli. (TIF) [file pcbi.1002091.s002.tif]

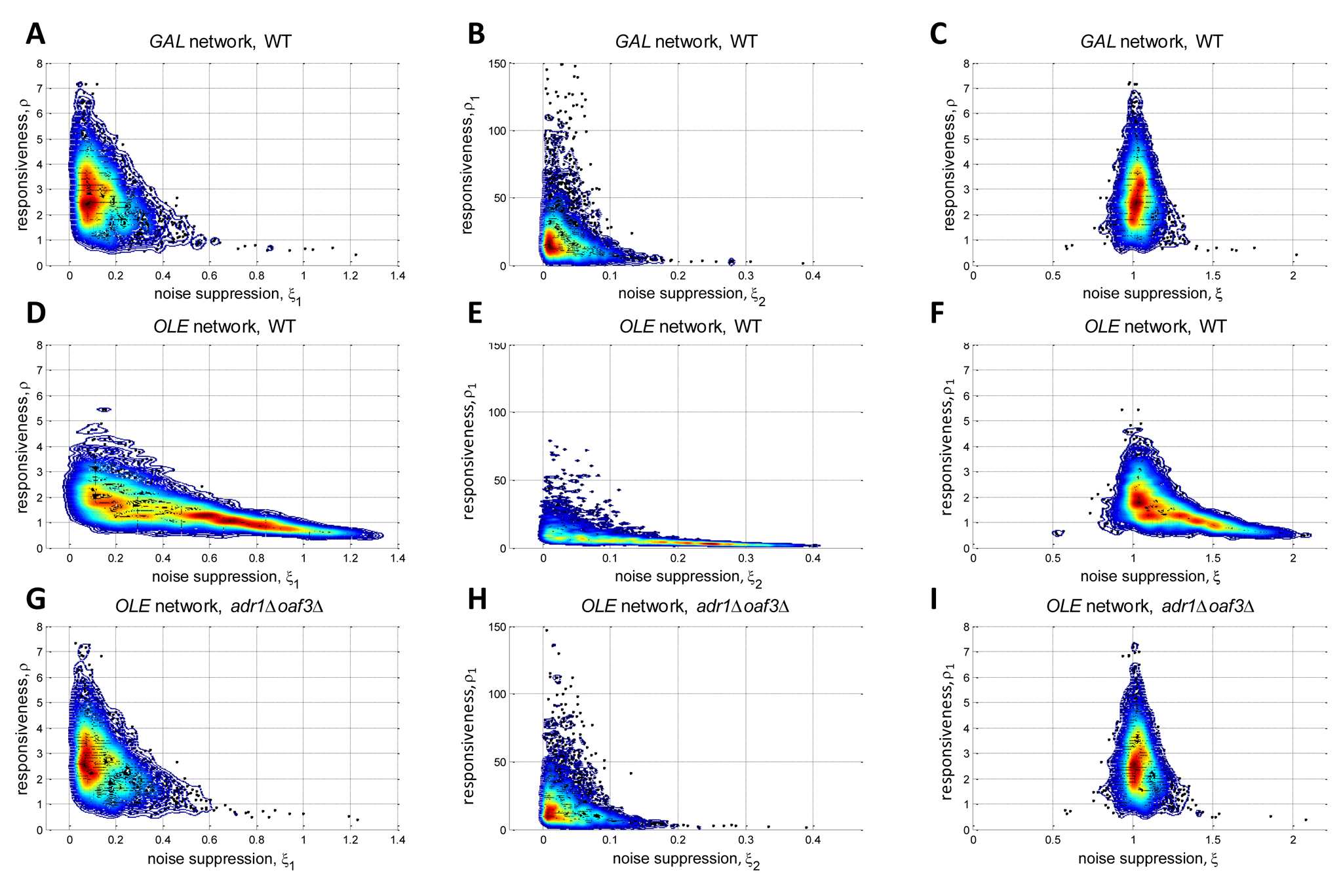

Supplement: Figure S3 — Responsiveness/noise suppression contour plots for (A, B, C) GAL, (D, E, F) WT-OLE, and (G, H, I) adr1Δoaf3Δ-OLE networks. Contour plots were calculated based on TFA characteristics of 3000 random stimuli/system responses (see Figure 2 in the main text). ξ1 denotes the symmetric Kullback-Leibler divergence between the distributions of the input and output spectrogram coefficient sums across all frequency bands. ξ2 denotes the Kolmogorov-Smirnov distance between the distributions of the input and output spectrogram coefficient sums across all frequency bands. ρ1 denotes the inverse Kolmogorov-Smirnov distance between the normalized total variation distributions of the input and output spectrogram coefficients across all frequency bands. The noise suppression and responsiveness statistics were calculated using the getHMforAllVarPrms MATLAB function (http://magnet.systemsbiology.net/tfa). (TIF) [file pcbi.1002091.s003.tif]

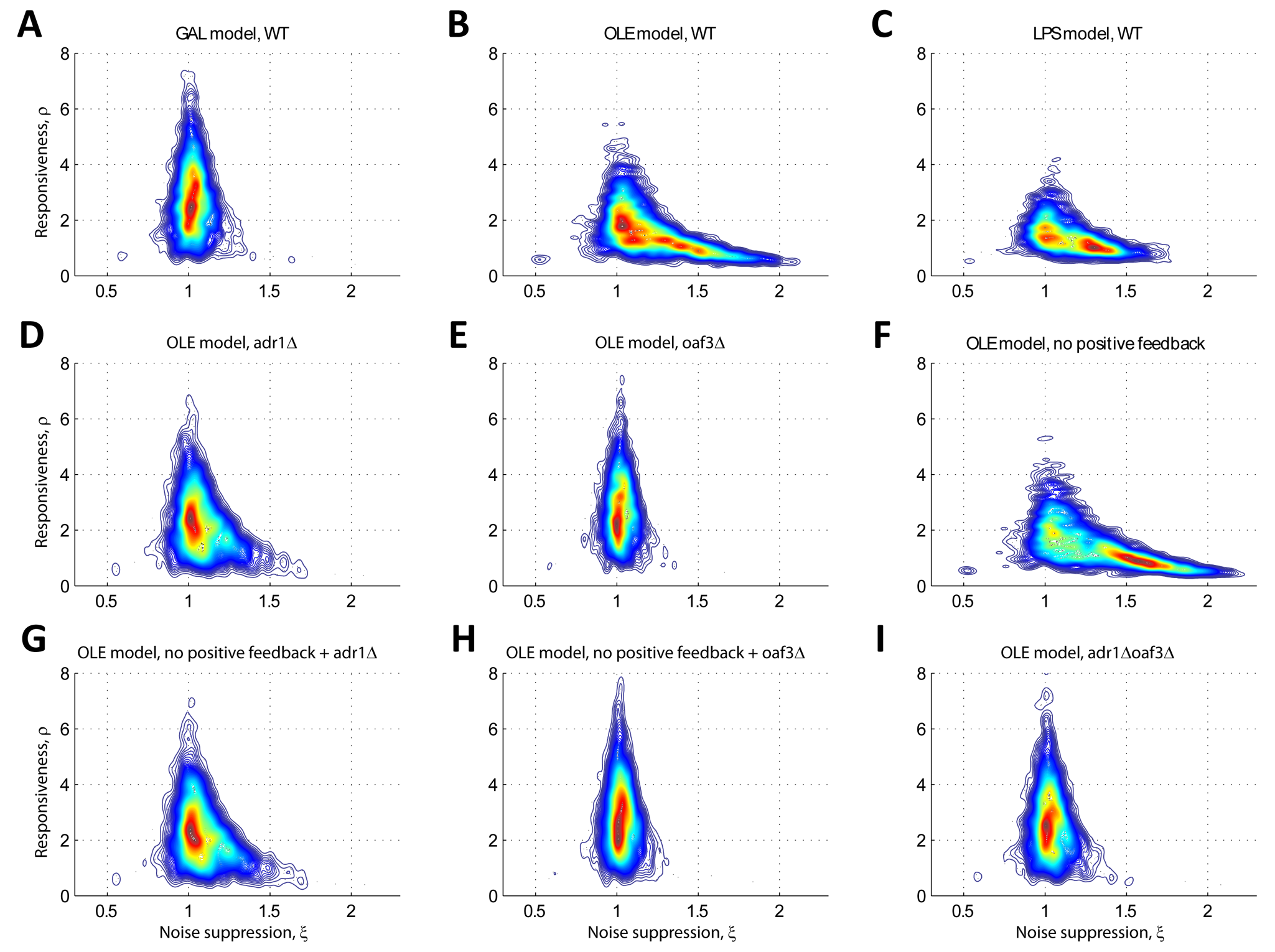

Supplement: Figure S4 — Responsiveness/noise suppression plots for (A) WT-GAL, (B) WT-OLE, (C) WT-LPS, (D) adr1Δ-OLE, (E) oaf3Δ-OLE, (F) “no positive feedback”-OLE, (G) “no positive feedback”-adr1Δ-OLE, (H) “no positive feedback”-oaf3Δ-OLE, (I) adr1Δoaf3Δ-OLE models. The ξ and ρ were calculated based on 3000 random time-varying stimuli and system responses. The contour plots were constructed using a bivariate Gaussian kernel density estimator (see Figure 2 in the main text and Figures S3, S5, S6 and S7). The “no positive feedback”-OLE model represents the OLE network where Pip2p does not upregulate its own gene PIP2 but upregulates its target genes. (TIF) [file pcbi.1002091.s004.tif]

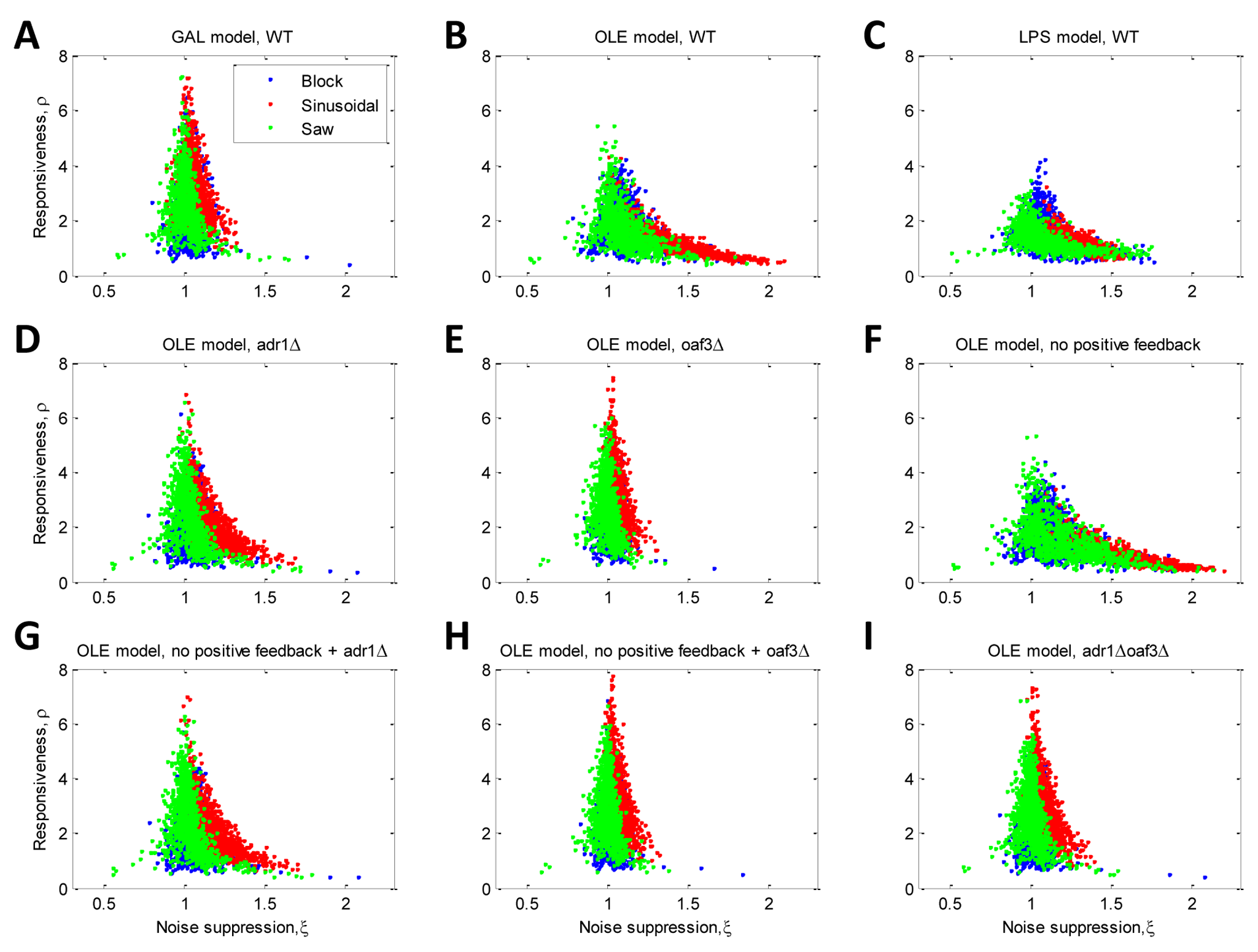

Supplement: Figure S5 — Colored scatter plots of the noise suppression and responsiveness statistics for (A) WT-GAL, (B) WT-OLE, (C) WT-LPS, (D) adr1Δ-OLE, (E) oaf3Δ-OLE, (F) “no positive feedback”-OLE, (G) “no positive feedback”-adr1Δ-OLE, (H) “no positive feedback”-oaf3Δ-OLE, (I) adr1Δoaf3Δ-OLE models. The ξ and ρ were calculated based on 3000 random time-varying stimuli and system responses (see Figure 2 in the main text and Figures S3, S4, S6 and S7). The color of the dots represents the type of stimuli applied to the networks. The blue, red and green dots represent “block”, sinusoidal, and “saw” signals, respectively. The “no positive feedback”-OLE model represents the OLE network where Pip2p does not upregulate its own gene PIP2 but upregulates its target genes. (TIF) [file pcbi.1002091.s005.tif]

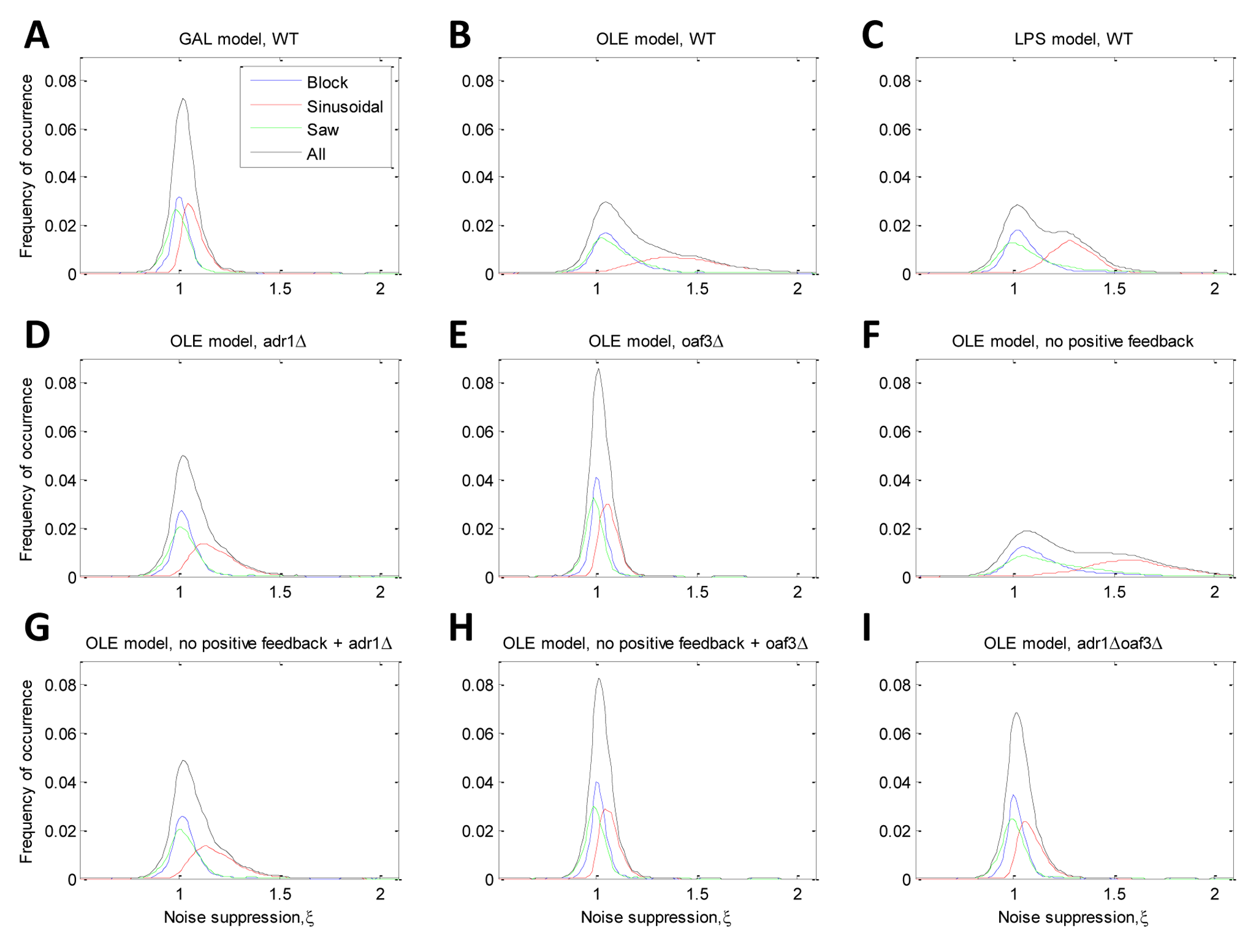

Supplement: Figure S6 — Distribution of the noise suppression characteristic for (A) WT-GAL, (B) WT-OLE, (C) WT-LPS, (D) adr1Δ-OLE, (E) oaf3Δ-OLE, (F) “no positive feedback”-OLE, (G) “no positive feedback”-adr1Δ-OLE, (H) “no positive feedback”-oaf3Δ-OLE, (I) adr1Δoaf3Δ-OLE models. The ξ was calculated based on 3000 random time-varying stimuli and system responses (see Figure 2 in the main text and Figures S3, S4, S5 and S7). The color of the density plots represents the type of stimuli applied to the networks. The blue, red, green and black density distributions represent random “block”, sinusoidal, “saw” and all together stimuli, respectively. The “no positive feedback”-OLE model represents the OLE network where Pip2p does not upregulate its own gene PIP2 but upregulates its target genes. (TIF) [file pcbi.1002091.s006.tif]

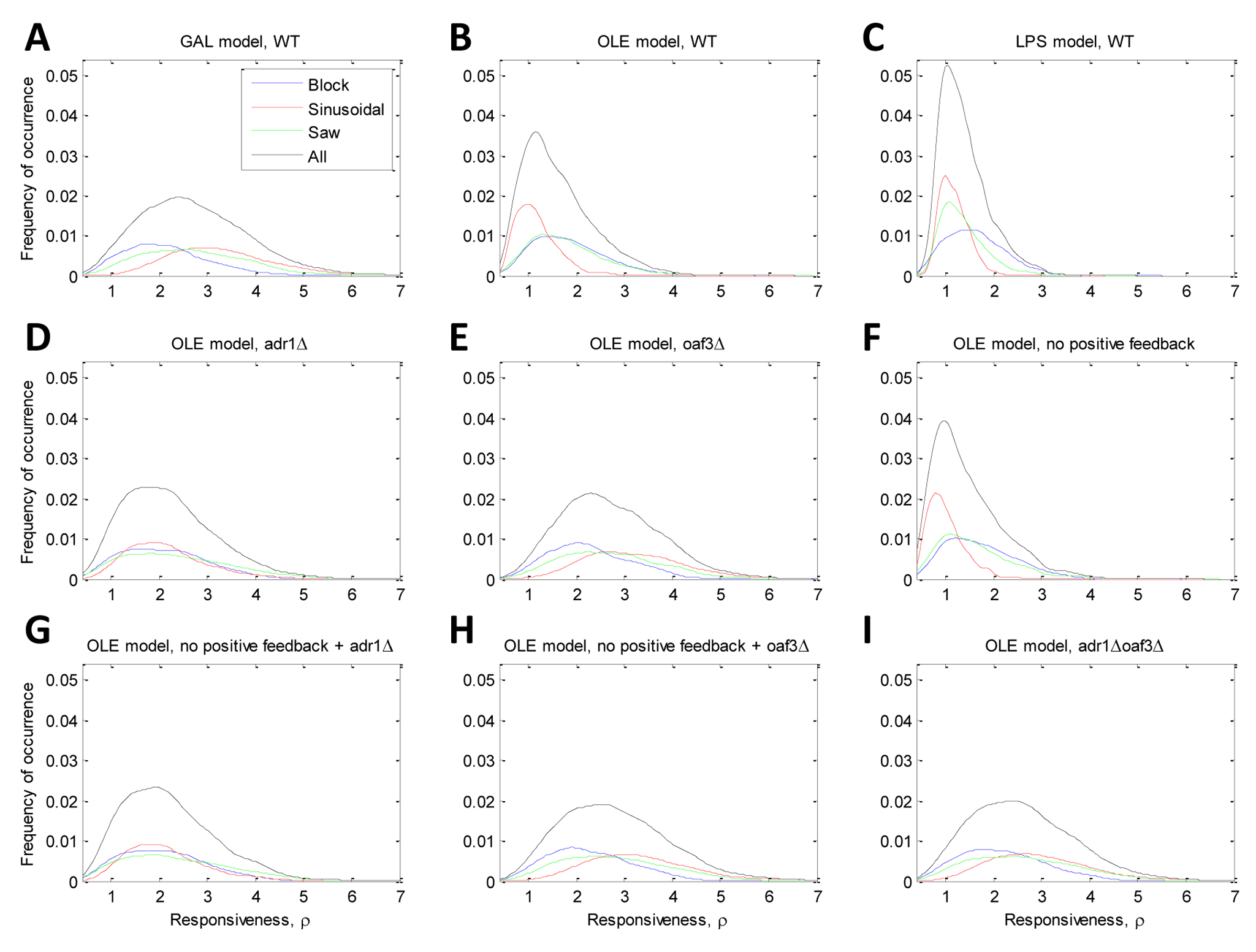

Supplement: Figure S7 — Distribution of the responsiveness characteristic for (A) WT-GAL, (B) WT-OLE, (C) WT-LPS, (D) adr1Δ-OLE, (E) oaf3Δ-OLE, (F) “no positive feedback”-OLE, (G) “no positive feedback”-adr1Δ-OLE, (H) “no positive feedback”-oaf3Δ-OLE, (I) adr1Δoaf3Δ-OLE models. The ρ was calculated based on 3000 random time-varying stimuli and system responses (see Figure 2 in the main text and Figures S3, S4, S5 and S6). The color of the density plots represents the type of stimuli applied to the networks. The blue, red, green and black density distributions represent random “block”, sinusoidal, “saw” and all together stimuli, respectively. The “no positive feedback”-OLE model represents the OLE network where Pip2p does not upregulate its own gene PIP2 but upregulates its target genes. (TIF) [file pcbi.1002091.s007.tif]

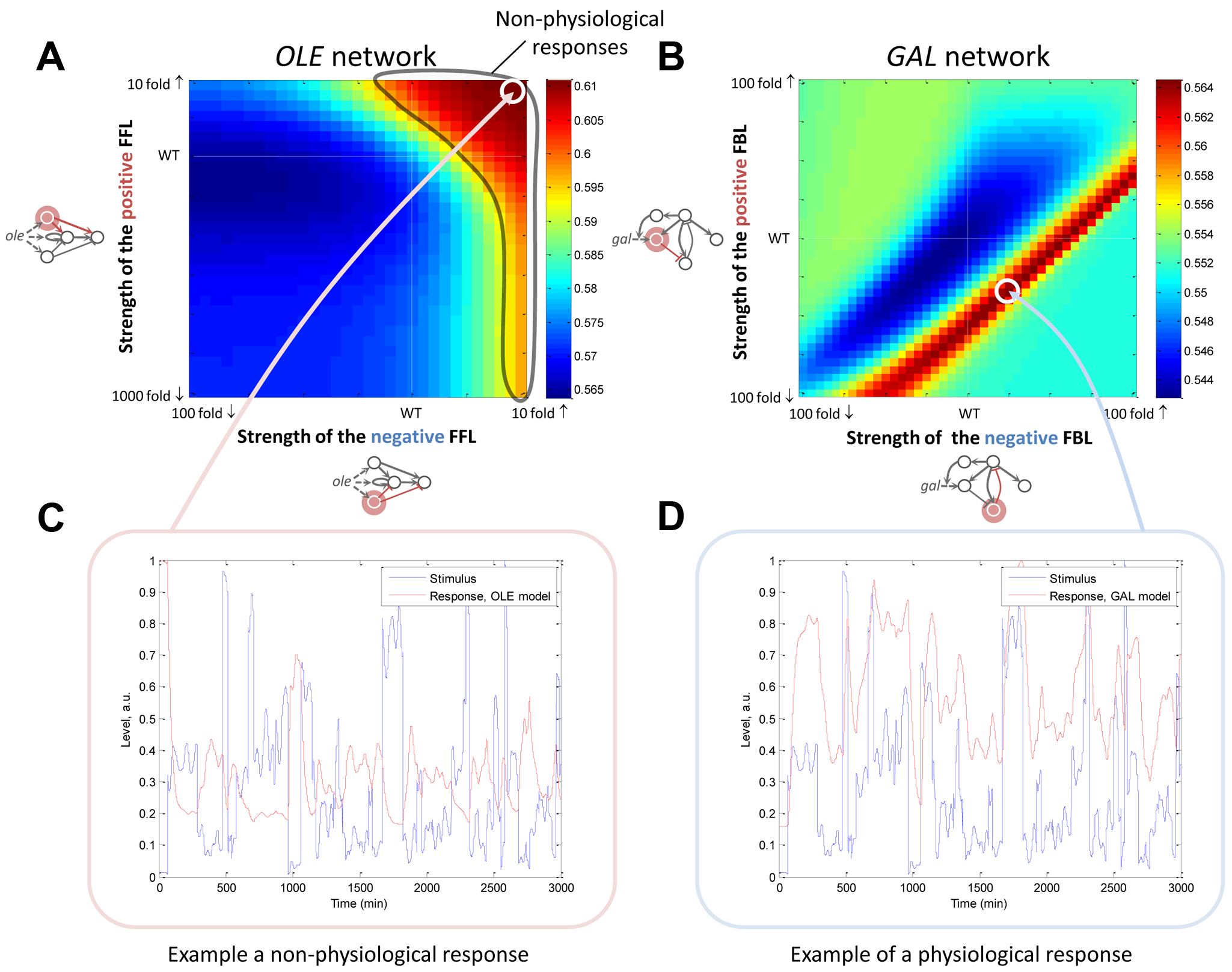

Supplement: Figure S8 — Physiological vs. non-physiological network responses. (A, B) The Euclidian distance between input and output derivatives of the OLE and GAL networks as a function of the strengths of positive and negative FFLs and FBLs, respectively. Each point on the heat maps represents the averaged Euclidian distance over 100 random and noisy stimuli (see Figure 3 in the main text). The strengths of the FFLs/FBLs are on a logarithmic scale. Non-physiological range of parameters for the OLE model is surrounded by the gray curve. (C) Example of a non-physiological response of the OLE model, which corresponds to the encircled area on the heat map (A). (D) Example of a physiological response of the GAL model, which corresponds to the encircled area on the heat map (B). There are no obvious non-physiological responses for the GAL model in the explored parameter space. (TIF) [file pcbi.1002091.s008.tif]

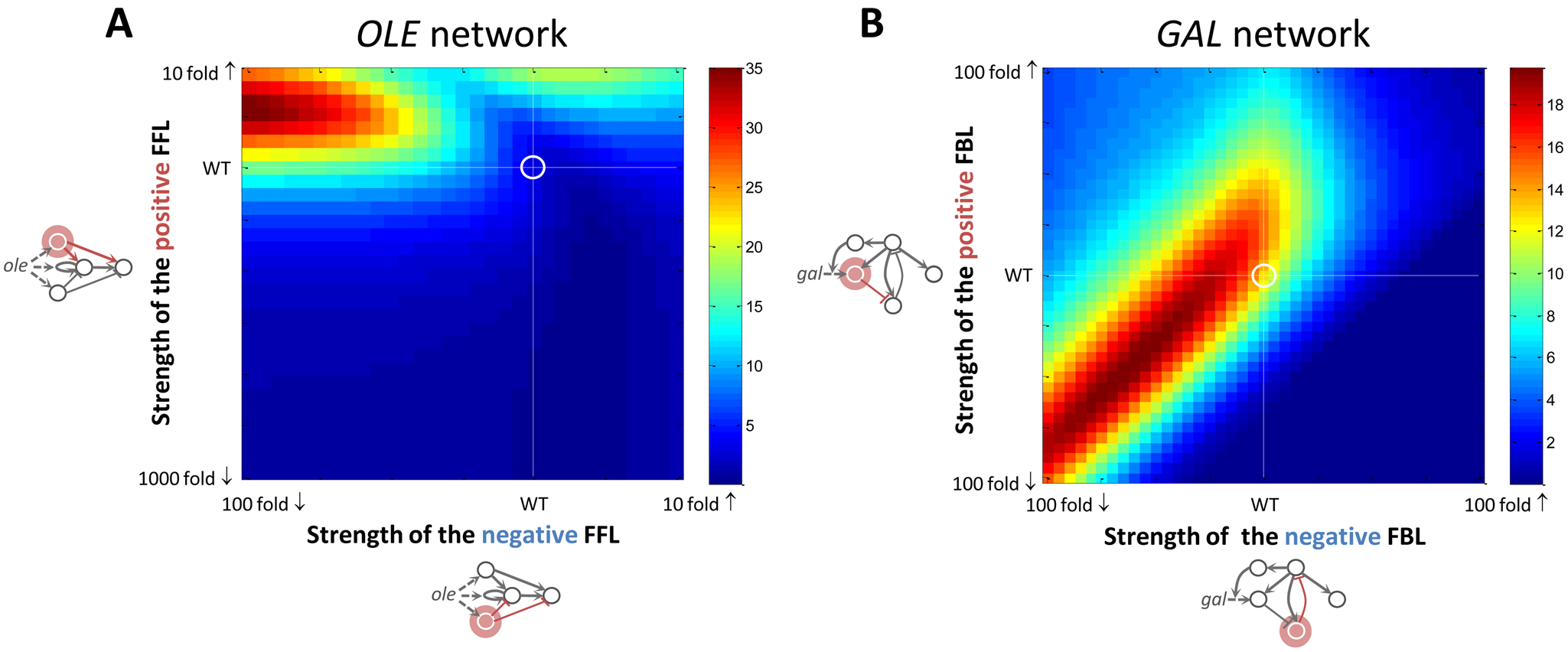

Supplement: Figure S9 — Amplitude of (A) OLE and (B) GAL network responses as a function of positive and negative FFL and FBL strengths, respectively. Each point on the heat maps represents the averaged amplitude over 100 random and noisy stimuli (see Figure 3 in the main text). The strengths of the FFLs/FBLs are on a logarithmic scale. White lines represent WT parameters and their encircled intersection is the WT network. (TIF) [file pcbi.1002091.s009.tif]

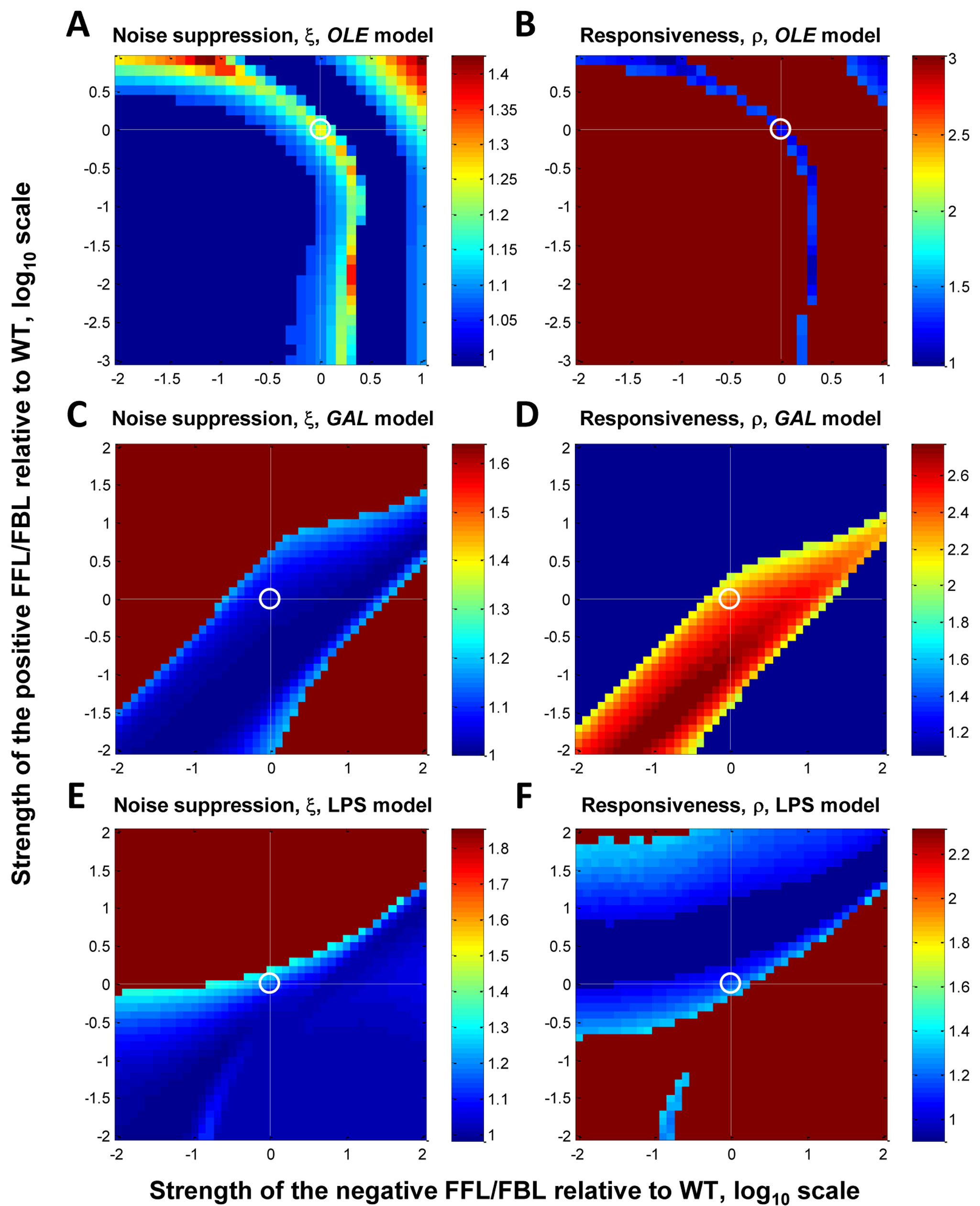

Supplement: Figure S10 — Noise suppression and responsiveness heat map areas within ±15% of the wild type ξ and ρ values for (A, B) OLE, (C, D) GAL and (E, F) LPS models, respectively. Heat map areas with ξ and ρ values below or above the threshold (±15% of WT values) are set to be equal to the minimum or the maximum value of the heat map, respectively. White lines represent WT parameters and their encircled' intersection is the WT network. (TIF) [file pcbi.1002091.s010.tif]

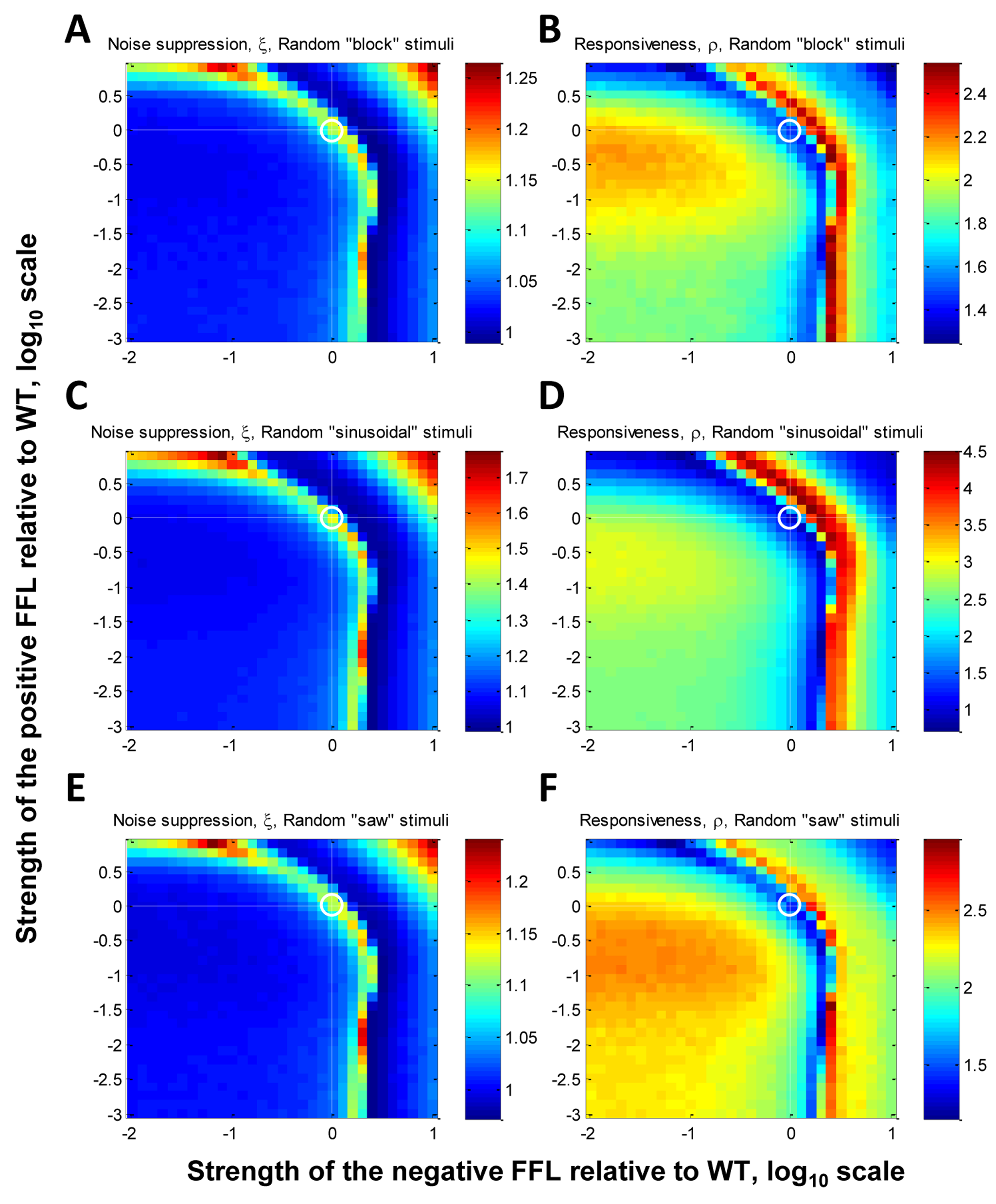

Supplement: Figure S11 — The noise suppression (ξ) and the responsiveness (ρ) of the OLE model as a function of the positive and negative FFL strengths. Each point on the heat maps represents the averaged ξ or ρ over (A, B) 33 random “block” or (C, D) 34 random sinusoidal or (E, F) 33 random “saw” stimuli. The strengths of the FFLs are on a logarithmic scale. White lines represent WT parameters and their encircled intersection is the WT network. (TIF) [file pcbi.1002091.s011.tif]

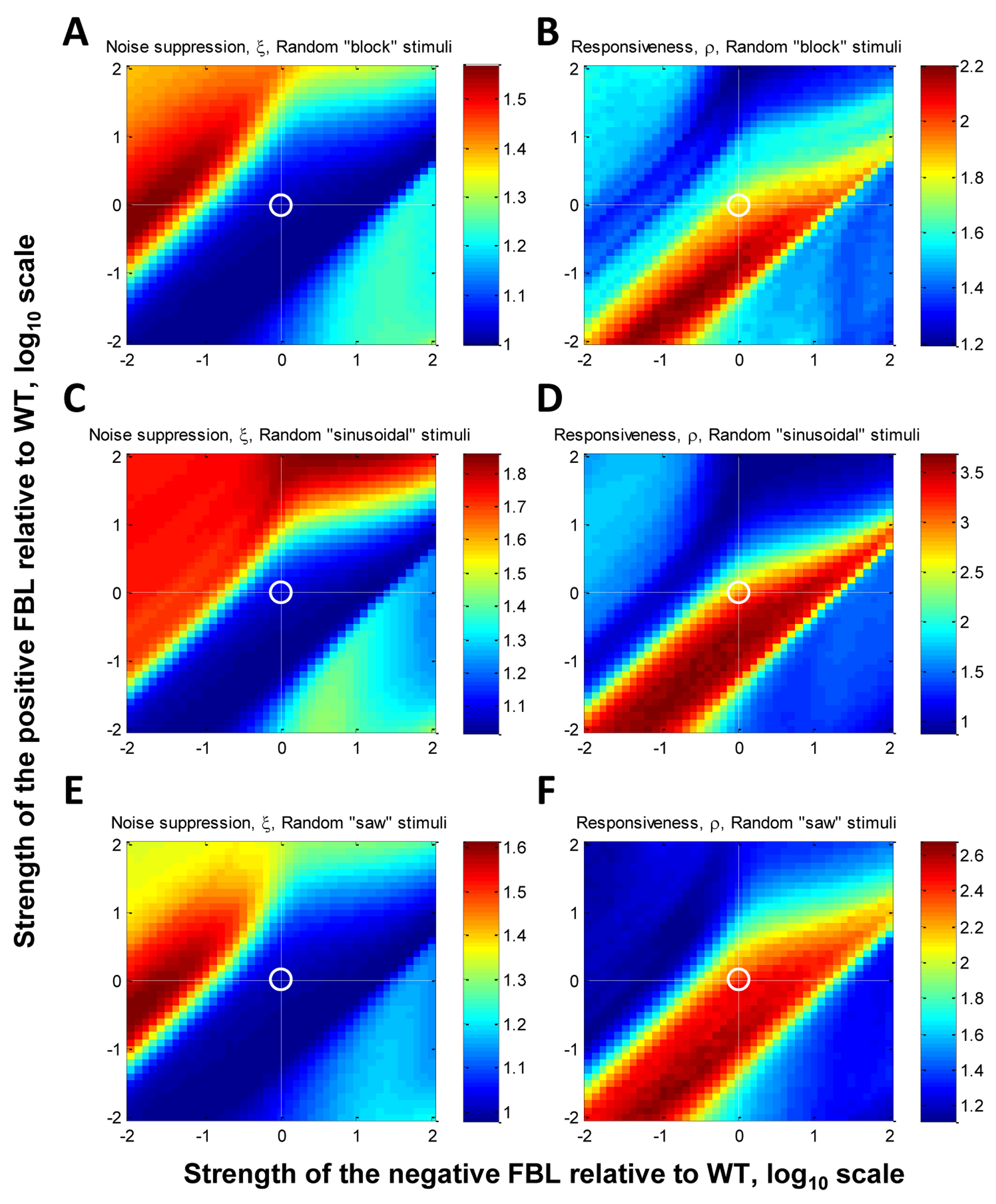

Supplement: Figure S12 — The noise suppression (ξ) and the responsiveness (ρ) of the GAL model as a function of the positive and negative FBL strengths. Each point on the heat maps represents the averaged ξ and ρ over (A, B) 33 random “block” or (C, D) 34 random sinusoidal or (E, F) 33 random “saw” stimuli. The strengths of the FBLs are on a logarithmic scale. White lines represent WT parameters and their encircled intersection is the WT network. (TIF) [file pcbi.1002091.s012.tif]

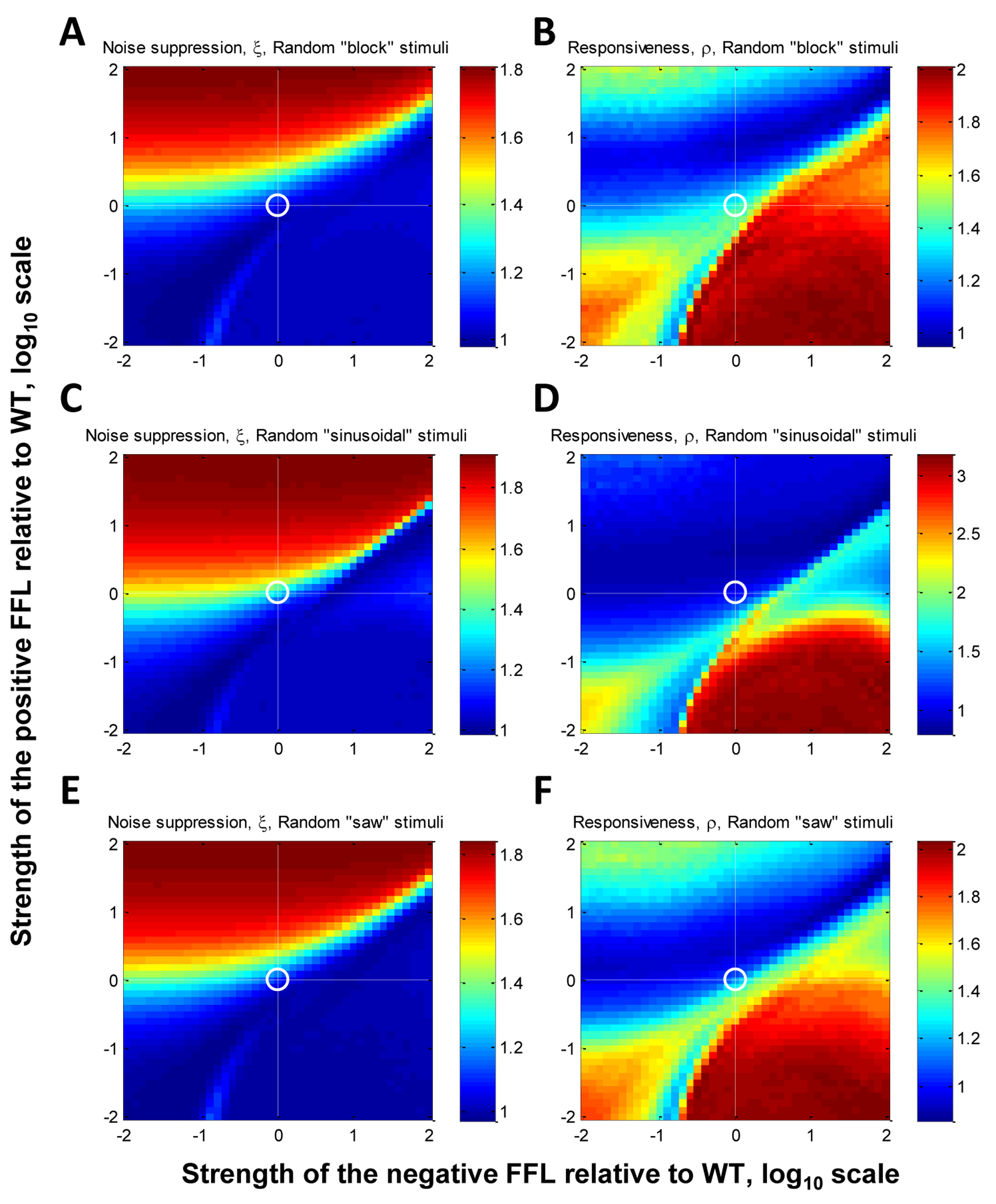

Supplement: Figure S13 — The noise suppression (ξ) and the responsiveness (ρ) of the LPS model as a function of the positive and negative FFL strengths. Each point on the heat maps represents the averaged ξ and ρ over (A, B) 33 random “block” or (C, D) 34 random sinusoidal or (E, F) 33 random “saw” stimuli. The strengths of the FFLs are on a logarithmic scale. White lines represent WT parameters and their encircled intersection is the WT network. (TIF) [file pcbi.1002091.s013.tif]
