# Supplementary material for: Trade-off between Responsiveness and Noise Suppression in Biomolecular System Responses to Environmental Cues
Source: PLoS Comput Biol. 2011 Jun 30;7(6):e1002091. doi: 10.1371/journal.pcbi.1002091 (PMC3127798; doi:10.1371/journal.pcbi.1002091)
Supplement: Table S1 — Equations defining the OLE network model. (DOC) [file pcbi.1002091.s014.doc]

**Table S1.** Equations defining the *OLE* network model.

| **Equation** | **Description** |
| --- | --- |
| $\frac{dr_{\gamma}(t)}{dt}=k_{i,r,\gamma}f_{r,\gamma}(p_{\gamma}(t))-k_{d,r,\gamma}r_{\gamma}(t),$ $\forall\gamma\in\{a,o,p,y,c\}$ | Gene expression |
| $\frac{dp_{\gamma}(t)}{dt}=k_{i,p,\gamma}r_{\gamma}(t)-k_{d,p,\gamma}p_{\gamma}(t),$ $\forall\gamma\in\{a,o,p,y,c\}$ | Protein expression |
| $\pi_{o}(t)=\frac{p_{o}O_{ic}}{K_{D,o}+O_{ic}}$ | Activation of Oaf1p by oleate (*π_o_* denotes activated Oaf1p) |
| $h(t)=\left(\frac{K_{D,h}}{2Q}\right)\left(1+\frac{Q}{K_{D,h}}(\pi_{o}+p_{p})-\sqrt{\left(1+\frac{Q}{K_{D,h}}(\pi_{o}+p_{p})\right)^{2}-4\left(\frac{Q}{K_{D,h}}\right)^{2}\pi_{o}p_{p}}\right)$ | Heterodimerization of activated Oaf1p with Pip2p (*h*(t) represents the concentration of Oaf1p-Pip2p heterodimer) |
| $\pi_{y}=\frac{p_{y}O_{ic}}{K_{D,o}+O_{ic}}$ | Activation of Oaf3p by oleate |
| $\pi_{a}=p_{a}\left(\varepsilon_{a}+(1-\varepsilon_{a})\cdot\frac{O_{ic}}{K_{M,a}+O_{ic}}\right)$ | Activation of Adr1p by oleate |
| $S=\frac{O_{ic}}{K_{M,s}+O_{ic}}$ | Activation of transcriptional response of *ADR1*, *OAF1*, and *OAF3* by oleate. |
| $f_{r,a}=\left(\frac{1}{r_{x,a}}\right)\left[r_{g,a}+S(r_{o,a}-r_{g,a})\right]$ | Fractional activity of *ADR1* gene |
| $f_{r,o}=\left(\frac{1}{r_{x,o}}\right)\left[r_{g,o}+S(r_{o,o}-r_{g,o})\right]$ | Fractional activity of *OAF1* gene |
| $f_{r,p}(t)=\frac{\varepsilon_{p}A_{p}+\frac{h}{K_{h}}+\frac{\pi_{a}}{K_{a}}+\frac{qh\pi_{a}}{K_{h}K_{a}}}{A_{p}+\frac{h}{K_{h}}+\frac{\pi_{a}}{K_{a}}+\frac{qh\pi_{a}}{K_{h}K_{a}}+\frac{\pi_{y}}{K_{y}}+\frac{h\pi_{y}}{K_{h}K_{y}}+\frac{\pi_{a}\pi_{y}}{K_{a}K_{y}}+\frac{h\pi_{a}\pi_{y}}{K_{h}K_{a}K_{y}}}$ | Fractional activity of *PIP2* gene |
| $f_{r,y}=\left(\frac{1}{r_{x,y}}\right)\left[r_{g,y}+S(r_{o,y}-r_{g,y})\right]$ | Fractional activity of *OAF3* gene |
| $f_{r,c}(t)=\frac{\varepsilon_{c}A_{c}+\frac{h}{K_{h}}+\frac{\pi_{a}}{K_{a}}+\frac{qh\pi_{a}}{K_{h}K_{a}}}{A_{c}+\frac{h(t)}{K_{h}}+\frac{\pi_{a}}{K_{a}}+\frac{qh\pi_{a}}{K_{h}K_{a}}+\frac{10\pi_{y}}{K_{y}}\left(1+\frac{h}{K_{h}}+\frac{\pi_{a}}{K_{a}}+\frac{h\pi_{a}}{K_{h}K_{a}}\right)}$ | Fractional activity of *CTA1* gene |
