# Supplementary material for: Trade-off between Responsiveness and Noise Suppression in Biomolecular System Responses to Environmental Cues
Source: PLoS Comput Biol. 2011 Jun 30;7(6):e1002091. doi: 10.1371/journal.pcbi.1002091 (PMC3127798; doi:10.1371/journal.pcbi.1002091)
Supplement: Table S2 — Dynamic variables of the OLE network model. (DOC) [file pcbi.1002091.s015.doc]

**Table S2.** Dynamic variables of the *OLE* network model.

| **Symbol** | **Species** | **Initial condition (molec/cell)** |
| --- | --- | --- |
| $\pi_{o}$ | Concentration of activated Oaf1p | calculated (Table S1) |
| $\pi_{a}$ | Concentration of activated Adr1p | calculated (Table S1) |
| $\pi_{y}$ | Concentration of activated Oaf3p | calculated (Table S1) |
| $h$ | Oaf1p-Pip2p heterodimer concentration | calculated (Table S1) |
| $p_{a}$ | Total concentration of Adr1p | (*r_d,a_/r_d,a_*)*p_d,a_*, (Table S3) |
| $p_{c}$ | Total concentration of Cta1p | (*r_d,c_/r_d,c_*)*p_d,c_*, (Table S3) |
| $p_{o}$ | Total concentration of Oaf1p | (*r_d,o_/r_d,o_*)*p_d,o_*, (Table S3) |
| $p_{y}$ | Total concentration of Oaf3p | (*r_d,y_/r_d,y_*)*p_d,y_*, (Table S3) |
| $p_{p}$ | Total concentration of Pip2p | (*r_d,p_/r_d,p_*)*p_d,p_*, (Table S3) |
| $r_{a}$ | mRNA concentration of *ADR1* | 3.78*r_d,a_*, (Table S3) |
| $r_{c}$ | mRNA concentration of *CTA1* | 11.5*r_d,c_*, (Table S3) |
| $r_{o}$ | mRNA concentration of *OAF1* | 3.0*r_d,o_*, (Table S3) |
| $r_{y}$ | mRNA concentration of *OAF3* | 3.43*r_d,y_*, (Table S3) |
| $r_{p}$ | mRNA concentration of *PIP2* | 2.13*r_d,a_*, (Table S3) |
