# Supplementary material for: Trade-off between Responsiveness and Noise Suppression in Biomolecular System Responses to Environmental Cues
Source: PLoS Comput Biol. 2011 Jun 30;7(6):e1002091. doi: 10.1371/journal.pcbi.1002091 (PMC3127798; doi:10.1371/journal.pcbi.1002091)
Supplement: Table S3 — Kinetic parameters and derived parameters used in the OLE network model. (DOC) [file pcbi.1002091.s016.doc]

**Table S3.** Kinetic parameters and derived parameters used in the *OLE* network model.

| **Symbol** | **Quantity** | **Estimated value** |
| --- | --- | --- |
| $\tau_{r,a}$ | *ADR1* mRNA half-life | 22 min |
| $\tau_{r,c}$ | *CTA1* mRNA half-life | 17 min |
| $\tau_{r,o}$ | *OAF1* mRNA half-life | 15 min |
| $\tau_{r,y}$ | *OAF3* mRNA half-life | 22 min |
| $\tau_{r,p}$ | *PIP2* mRNA half-life | 22 min |
| $\tau_{p,a}$ | Adr1 protein half-life | 180 min |
|  | Cta1 protein half-life (oleate) | 480 min |
| $\tau_{p,o}$ | Oaf1 protein half-life | 54.6 min |
|  | Oaf3 protein half-life | 144 min |
| $\tau_{p,p}$ | Pip2 protein half-life | ~40 min |
|  | *ADR1* mRNA conc, glucose | 0.25 molec/cell |
| $r_{o,c}$ | *CTA1* mRNA conc, oleate | 76.7 molec/cell |
| $r_{d,o}$ | *OAF1* mRNA conc, glucose | 0.6 molec/cell |
|  | *OAF3* mRNA conc, glucose | 0.4 molec/cell |
| $r_{d,p}$ | *PIP2* mRNA conc, glucose | 0.3 molec/cell |
| $r_{g,a}/r_{d,a}$ | *ADR1* mRNA ratio, glycerol/glucose | 3.78 |
| $r_{g,c}/r_{d,c}$ | *CTA1* mRNA ratio, glycerol/glucose | 11.5 |
| $r_{g,o}/r_{d,o}$ | *OAF1* mRNA ratio, glycerol/glucose | 0.9794 |
| $r_{g,y}/r_{d,y}$ | *OAF3* mRNA ratio, glycerol/glucose | 1.43 |
| $r_{g,p}/r_{d,p}$ | *PIP2* mRNA ratio, glycerol/glucose | 2.13 |
| $r_{o,a}/r_{d,a}$ | *ADR1* mRNA ratio, oleate/glucose | 4.75 |
| $r_{o,c}/r_{d,c}$ | *CTA1* mRNA ratio, oleate/glucose | 79.8 |
| $r_{o,o}/r_{d,o}$ | *OAF1* mRNA ratio, oleate/glucose | 0.9 |
| $r_{o,y}/r_{d,y}$ | *OAF3* mRNA ratio, oleate/glucose | 1.35 |
| $r_{o,p}/r_{d,p}$ | *PIP2* mRNA ratio, oleate/glucose | 7.4 |
| $r_{g,c}/r_{g,c,\Delta\text{adr1}}$ | *CTA1* mRNA ratio, non-induced, WT/(Δadr1) | 3.1 |
| $r_{g,p}/r_{g,p,\Delta\text{adr1}}$ | *PIP2* mRNA ratio, non-induced, WT/(Δadr1) | 1.3 |
| $p_{d,a}$ | total Adr1p concentration, glucose | ~50 molec/cell |
| $p_{d,c}$ | total Cta1p concentration, glucose | 623 molec/cell |
| $p_{d,o}$ | total Oaf1p concentration, glucose | 92.3 molec/cell |
| $p_{d,y}$ | total Oaf1p concentration, glucose | 149 molec/cell |
| $p_{d,p}$ | total Pip2p concentration, glucose | ~50 molec/cell |
| $p_{g,a}$ | total Adr1p concentration, glycerol | 189 molec/cell |
| $p_{g,c}$ | total Cta1p concentration, glycerol | 2,242 molec/cell |
| $p_{g,o}$ | total Oaf1p concentration, glycerol | 90.4 molec/cell |
| $p_{g,y}$ | total Oaf3p concentration, glycerol | 213 molec/cell |
| $p_{g,p}$ | total Pip2p concentration, glycerol | 107 molec/cell |
| $r_{x,a}/r_{d,a}$ | *ADR1* mRNA max expression ratio (relative to glucose) | 15.0 |
| $r_{x,c}/r_{d,c}$ | *CTA1* mRNA max expression ratio (relative to glucose) | 97.1 |
| $r_{x,o}/r_{d,o}$ | *OAF1* mRNA max expression ratio (relative to glucose) | 5.9 |
| $r_{x,y}/r_{d,y}$ | *OAF3* mRNA max expression ratio (relative to glucose) | 3.2 |
| $r_{x,p}/r_{d,p}$ | *PIP2* mRNA max expression ratio  (relative to glucose) | 20 |
| $k_{d,r,a}$ | *ADR1* mRNA rate of degradation | 0.0315 min-1 |
| $k_{d,r,c}$ | *CTA1* mRNA rate of degradation | 0.0408 min-1 |
| $k_{d,r,o}$ | *OAF1* mRNA rate of degradation | 0.0462 min-1 |
| $k_{d,r,y}$ | *OAF3* mRNA rate of degradation | 0.0315 min-1 |
| $k_{d,r,p}$ | *PIP2* mRNA rate of degradation | 0.0315 min-1 |
| $k_{d,p,a}$ | Adr1p rate of degradation | 0.0039 min-1 |
| $k_{d,p,c}$ | Cta1p rate of degradation | 0.00048 min-1 |
| $k_{d,p,o}$ | Oaf1p rate of degradation | 0.0127 min-1 |
| $k_{d,p,y}$ | Oaf3p rate of degradation | 0.00481 min-1 |
| $k_{d,p,p}$ | Pip2p rate of degradation | 0.0173 min-1 |
| $k_{i,r,a}$ | *ADR1* max rate of initiation of transcription | 0.118 (molec/cell) min-1 |
| $k_{i,r,c}$ | *CTA1* max rate of initiation of transcription | 4.12 (molec/cell) min-1 |
| $k_{i,r,o}$ | *OAF1* max rate of initiation of transcription | 0.164 (molec/cell) min-1 |
| $k_{i,r,y}$ | *OAF3* max rate of initiation of transcription | 0.0403 (molec/cell) min-1 |
| $k_{i,r,p}$ | *PIP2* max rate of initiation of transcription | 0.189 (molec/cell) min-1 |
| $k_{i,p,a}$ | *ADR1* rate of initiation of translation, per mRNA | 0.770 min-1 |
| $k_{i,p,c}$ | *CTA1* rate of initiation of translation, per mRNA | 0.865 min-1 |
| $k_{i,p,o}$ | *OAF1* rate of initiation of translation, per mRNA | 1.95 min-1 |
| $k_{i,p,y}$ | *OAF3* rate of initiation of translation, per mRNA | 1.79 min-1 |
| $k_{i,p,p}$ | *PIP2* rate of initiation of translation, per mRNA | 2.88 min-1 |
| $\varepsilon_{c}$ | Constitutive term in fractional activity of *CTA1* | 0.0382 |
| $\varepsilon_{p}$ | Constitutive term in fractional activity of *PIP2* | 0.0819 |
| *N* | Avogadro’s constant | 6.022 × 1023 molec/mol |
| *A* | cell cross-sectional area, on oleate | 11.8 m2­ |
| *V*c | cell volume, on oleate | 3 × 10^-14^ L |
| *V*n | nuclear volume, on oleate | 2.1 × 10^-15^ L |
| *Q* | Conversion between molecules/cell and molar, for a species that is localized to the nucleus | 7.9 × 10^-10^ M/(molec/cell) |
| *Moa* | Molecular weight of oleic acid | 282.46 g/mol |
| *H* | Conversion between % and molar, for oleic acid | 0.0354 M / (1 g / 100 mL) |
| *Oic* | Concentration of intracellular oleic acid | 0 – 4.25×10^-6^ M |
| $K_{D,o}$ | Dissociation constant for Oaf1p activation by free oleic acid | 1.65 × 10^-8^ M |
| *q* | Cooperativity of Adr1p and Oaf1p-Pip2p binding to DNA | 2.5 |
| *r* | Asymptotic ratio of intracellular to extracellular free fatty acid | 0.01 |
| $\varepsilon_{a}$ | Minimum fractional activity of Adr1p protein | 0.193 |
| $K_{h}$ | Equilibrium constant for Oaf1p-Pip2p binding to promoter | 8.19 molec/cell |
| $K_{a}$ | Equilibrium constant for Adr1p binding to promoter | 19.5 molec/cell |
| *Ac* | Activation constant for *CTA1* | 28 |
| *Ap* | Activation constant for *PIP2* | 197.3 |
| $K_{y}$ | Equilibrium constant for Oaf3p binding to promoter | 500 molec/cell |
| $K_{M,s}$ | Michaelis constant for oleate transcriptional response | 7.61 × 10^-6^ M |
| $K_{D,h}$ | Dissociation constant for Oaf1p-Pip2p heterodimer formation | 3.89 × 10^-6^ M |
| $K_{M,a}$ | Michaelis constant for activation of Adr1p | 8.42 × 10^-5^ M |
