# Supplementary material for: Trade-off between Responsiveness and Noise Suppression in Biomolecular System Responses to Environmental Cues
Source: PLoS Comput Biol. 2011 Jun 30;7(6):e1002091. doi: 10.1371/journal.pcbi.1002091 (PMC3127798; doi:10.1371/journal.pcbi.1002091)
Supplement: Table S4 — Mean, standard deviation (SD) and coefficient of variation (CV) values of the noise suppression (ξ) and responsiveness (ρ) characteristics for the WT-GAL, WT-OLE, WT-LPS, adr1Δ-OLE, oaf3Δ-OLE, “no positive feedback”-OLE, “no positive feedback”-adr1Δ-OLE, “no positive feedback”-oaf3Δ-OLE and adr1Δoaf3Δ-OLE models calculated for 1000 “block”, 1000 “saw”, and 1000 sinusoidal random signals (see Figure 2 in the main text and Figures S3, S4, S5, S6 and S7). (DOC) [file pcbi.1002091.s017.doc]

**Table S4.** Mean, standard deviation (SD) and coefficient of variation (CV) values of the noise suppression (ξ) and responsiveness (ρ) characteristics for the WT-*GAL,* WT-*OLE*, WT-LPS, *adr1*Δ-*OLE*, *oaf3*Δ-*OLE*, “no positive feedback”-*OLE*, “no positive feedback”-*adr1*Δ-*OLE*, “no positive feedback”-*oaf3*Δ-*OLE* and *adr1*Δ*oaf3*Δ-*OLE* models calculated for 1000 "block", 1000 “saw”, and 1000 sinusoidal random signals (see Figure 2 in the main text and Figures S3, S4, S5, S6 and S7).

| **Model** | **Type of random stimuli** | **ξ value, mean** | **ξ value, SD** | **ξ value, CV** | **ρ value, mean** | **ρ value, SD** | **ρ value, CV** |
| --- | --- | --- | --- | --- | --- | --- | --- |
| WT-*GAL* | Block | 1.016 | 0.065 | 0.064 | 2.138 | 0.862 | 0.403 |
|  | Sinusoidal | 1.078 | 0.057 | 0.053 | 3.298 | 1.082 | 0.328 |
|  | Saw | 1.001 | 0.079 | 0.079 | 2.720 | 1.077 | 0.396 |
|  | All | 1.032 | 0.076 | 0.073 | 2.732 | 1.119 | 0.409 |
| WT-*OLE* | Block | 1.100 | 0.122 | 0.111 | 1.831 | 0.722 | 0.395 |
|  | Sinusoidal | 1.435 | 0.200 | 0.139 | 1.172 | 0.451 | 0.385 |
|  | Saw | 1.095 | 0.135 | 0.123 | 1.807 | 0.799 | 0.442 |
|  | All | 1.208 | 0.222 | 0.184 | 1.608 | 0.741 | 0.461 |
| WT-LPS | Block | 1.078 | 0.133 | 0.124 | 1.609 | 0.607 | 0.377 |
|  | Sinusoidal | 1.293 | 0.103 | 0.080 | 1.176 | 0.307 | 0.261 |
|  | Saw | 1.091 | 0.173 | 0.159 | 1.356 | 0.462 | 0.341 |
|  | All | 1.153 | 0.171 | 0.148 | 1.382 | 0.508 | 0.367 |
| *adr1*Δ-*OLE* | Block | 1.038 | 0.083 | 0.080 | 2.089 | 0.864 | 0.414 |
|  | Sinusoidal | 1.185 | 0.113 | 0.095 | 2.189 | 0.923 | 0.422 |
|  | Saw | 1.030 | 0.104 | 0.100 | 2.368 | 1.100 | 0.465 |
|  | All | 1.084 | 0.123 | 0.114 | 2.215 | 0.974 | 0.440 |
| *oaf3*Δ-*OLE* | Block | 1.015 | 0.046 | 0.046 | 2.257 | 0.812 | 0.360 |
|  | Sinusoidal | 1.068 | 0.047 | 0.044 | 3.215 | 1.058 | 0.329 |
|  | Saw | 0.991 | 0.057 | 0.057 | 2.699 | 1.002 | 0.371 |
|  | All | 1.024 | 0.060 | 0.058 | 2.719 | 1.038 | 0.382 |
| “no positive feedback”-*OLE* | Block | 1.153 | 0.179 | 0.155 | 1.712 | 0.713 | 0.417 |
|  | Sinusoidal | 1.582 | 0.205 | 0.130 | 1.014 | 0.405 | 0.399 |
|  | Saw | 1.194 | 0.212 | 0.178 | 1.613 | 0.779 | 0.483 |
|  | All | 1.307 | 0.277 | 0.212 | 1.45 | 0.723 | 0.498 |
| “no positive feedback”-*adr1*Δ-*OLE* | Block | 1.040 | 0.085 | 0.082 | 2.087 | 0.864 | 0.414 |
|  | Sinusoidal | 1.191 | 0.117 | 0.098 | 2.177 | 0.933 | 0.429 |
|  | Saw | 1.034 | 0.107 | 0.103 | 2.375 | 1.110 | 0.468 |
|  | All | 1.087 | 0.127 | 0.116 | 2.213 | 0.982 | 0.444 |
| “no positive feedback”-*oaf3*Δ-*OLE* | Block | 1.018 | 0.054 | 0.053 | 2.284 | 0.869 | 0.380 |
|  | Sinusoidal | 1.073 | 0.051 | 0.047 | 3.357 | 1.155 | 0.344 |
|  | Saw | 0.997 | 0.062 | 0.062 | 2.810 | 1.094 | 0.389 |
|  | All | 1.029 | 0.064 | 0.062 | 2.812 | 1.133 | 0.403 |
| *adr1*Δ*oaf3*Δ-*OLE* | Block | 1.020 | 0.067 | 0.066 | 2.195 | 0.876 | 0.399 |
|  | Sinusoidal | 1.095 | 0.065 | 0.060 | 3.036 | 1.145 | 0.377 |
|  | Saw | 1.004 | 0.079 | 0.079 | 2.620 | 1.085 | 0.414 |
|  | All | 1.039 | 0.081 | 0.078 | 2.613 | 1.095 | 0.419 |
