# Supplementary material for: Trade-off between Responsiveness and Noise Suppression in Biomolecular System Responses to Environmental Cues
Source: PLoS Comput Biol. 2011 Jun 30;7(6):e1002091. doi: 10.1371/journal.pcbi.1002091 (PMC3127798; doi:10.1371/journal.pcbi.1002091)
Supplement: Table S5 — Mean values of the noise suppression (ξ) and responsiveness (ρ) WT characteristics for the GAL, OLE and LPS models calculated for 33 “block”, 33 “saw”, and 34 sinusoidal random signals (see Figures 3 and 4 in the main text). (DOC) [file pcbi.1002091.s018.doc]

**Table S5.** Mean values of the noise suppression (ξ) and responsiveness (ρ) WT characteristics for the *GAL*, *OLE* and LPS models calculated for 33 "block", 33 “saw”, and 34 sinusoidal random signals (see Figures 3 and 4 in the main text).

| **Model** | **Type of random stimuli** | **WT ξ value** | **WT ρ value** |
| --- | --- | --- | --- |
| *GAL* | Block | 1.017 | 1.857 |
|  | Sinusoidal | 1.082 | 2.910 |
|  | Saw | 1.002 | 2.411 |
|  | All | 1.037 | 2.357 |
| *OLE* | Block | 1.138 | 1.456 |
|  | Sinusoidal | 1.464 | 0.974 |
|  | Saw | 1.118 | 1.413 |
|  | All | 1.249 | 1.226 |
| LPS | Block | 1.070 | 1.383 |
|  | Sinusoidal | 1.293 | 1.075 |
|  | Saw | 1.104 | 1.168 |
|  | All | 1.161 | 1.190 |
