# Supplementary material for: Trade-off between Responsiveness and Noise Suppression in Biomolecular System Responses to Environmental Cues
Source: PLoS Comput Biol. 2011 Jun 30;7(6):e1002091. doi: 10.1371/journal.pcbi.1002091 (PMC3127798; doi:10.1371/journal.pcbi.1002091)
Supplement: Text S1 — This document contains additional supplemental information on the generation of random stimuli, mathematical models and computational methods used in this work. (DOC) [file pcbi.1002091.s019.doc]

*Supporting Information*

**Trade-off between responsiveness and noise suppression in biomolecular system responses to environmental cues**

Alexander V. Ratushny, Ilya Shmulevich, John D. Aitchison

**Generation of random stimuli**

While intuitively we expect that most natural stimuli would be well approximated with forms of a sinusoid, other forms of stimuli are typical in natural systems. Thus, random sinusoidal, “block” and “saw” shaped stimuli were used to systematically explore the dynamical properties of the *OLE*, *GAL* and LPS-induced networks. The particular shape of each stimulus was generated randomly. For random “block” and “saw” stimuli (Fig. S2*A* and *B*), two uniformly distributed arrays of random numbers in the interval [0, 1] were generated. The ascending order of the first array corresponds to the time points of jumps in the stimulus. The second array reflects the differences between the successive signal levels at the jump locations in the stimulus. The size of the arrays was varied between 3 and 60, reflecting different numbers of jumps in the stimulus.

Random sinusoidal stimuli (Fig. S2*C*) were generated using the expression

$$S(t)=k_{1,1}F(2\pi f_{1}t)+k_{2,1}F(2\pi f_{2}t)+k_{3,1}F(2\pi f_{3}t)+k_{4,1}F(2\pi f_{4}t),$$

where $F(\cdot)$ is either the sin or cos function, each chosen randomly,

$$f_{1}=20k_{1,2}(1+k_{1,3}t)$$

$$f_{2}=20k_{2,2}(1-k_{2,3}t)$$

$$f_{3}=20k_{3,2}(1+k_{3,3}t^{2})$$

$$f_{4}=20k_{4,2}(1-k_{4,3}t^{2})$$

and *k_i,j_* are random uniformly distributed parameters ($k_{i,j}\in\text{[}0, 1\text{]}, i=1,\ldots,4, j=1,\ldots,3$).

Gaussian white noise, smoothed by an equiripple low-pass filter (passband frequency = 0.2; stopband frequency = 0.4; passband ripple = 0.0575; stopband attenuation = 0.0001), and scaled by $0.2\times\sqrt{S(t)},$where $S(t)$ is the signal, was added to each stimulus. Thus, the noise power is proportional to the signal.

The amplitude range of the generated random time variant stimuli was scaled so that the maximum amplitude of the stimulus for the *GAL* network corresponds to 11.1 mM of external galactose, the maximum amplitude of the stimulus for the *OLE* network corresponds to 4.25×10^-6^ M of intracellular oleate, and the maximum amplitude of the stimulus for the LPS-induced network corresponds to 1,500 molecules/cell of the nuclear NFkB. The duration of the generated random time variant stimuli was scaled to be equal to 3000 min.

Random stimuli were generated using the *precalcInputSignals* MATLAB function (<http://magnet.systemsbiology.net/tfa>).

**Mathematical models**

Kinetic models of the *OLE*, *GAL* and LPS-induced networks are presented below. The ordinary differential equation (ODE) kinetic models of the *OLE* and *GAL* networks and the delay differential equation (DDE) kinetic model of the LPS-induced network were solved using the standard ODE and DDE solvers, respectively, in MATLAB.

***Kinetic model of the OLE network***

Simplified version of the ODE model of the *OLE* network (1) was used. The five genes in the model are abbreviated by the subscripts as follows: *ADR1* (*a*), *OAF1* (*o*), *PIP2* (*p*), *OAF3* (*y*), and *CTA1* (*c*). Equations, dynamic variables and kinetic parameters of the model are presented in Tables S1, S2 and S3, respectively.

*Oic* was varied as a stimulus in the model (Fig. S2, the y-axis unit of the figure plots corresponds to 4.25×10^-6^ M of *Oic*). To generate the heat maps presented in Fig. 3*A* and *B* in the main text, the model parameters *K_a_* (strength of the positive FFL) and *K_y_* (strength of the negative FFL) were varied.

*OLE* network model responses (the target gene expression profiles) to random noisy stimuli for the set of parameters specified by *K_a_* and *K_y_* were calculated using the *oleModelMulpRun* MATLAB function (<http://magnet.systemsbiology.net/tfa>).

***Kinetic model of the GAL network***

The ODE model of the *GAL* network published by Ramsey *et al.* (2) was used. Equations for the *GAL* network model are available at <http://magnet.systemsbiology.net/galnoise>. *G_ex_* was varied as a stimulus in the model (Fig. S2, the y-axis unit of the figure plots corresponds to 11.1 mM (2.39×10^8^ molec/cell) of *G_ex_*). To generate the heat maps presented in Fig. 3*C* and *D* in the main text, the model parameters *k_ir_*_,gal3_ (mimicking the strength of the positive FBL) and *k_ir_*_,gal80_ (mimicking the strength of the negative FBL) were varied.

*GAL* network model responses (the target gene expression profiles) to random noisy stimuli for the set of parameters specified by *k_ir_*_,gal3_ and *k_ir_*_,gal80_ were calculated using *galModelMulpRun* MATLAB function (<http://magnet.systemsbiology.net/tfa>).

***Kinetic model of the LPS-induced regulatory network***

The DDE model of the LPS-induced regulatory network published by Litvak *et al.* (3) was used. *p*_0_ was varied as a stimulus in the model. The model parameters *w*_12_, *w*_13_, *w*_22_, *w*_03_ and *w*_23_ were multiplied by *k*_FFL_^+^ (mimicking the strength of the positive FFL) and *w*_12_ and *w*_13_ were multiplied by *k*_FFL_^-^ (mimicking the strength of the negative FFL). To generate the heat maps presented in Fig. 4*B* and *C* in the main text, *k*_FFL_^+^ and *k*_FFL_^-^ were varied.

LPS-induced network model responses (the target gene expression profiles) to random noisy stimuli for the set of parameters specified by *k*_FFL_^+^ and *k*_FFL_^-^ were calculated using *cebpdModelMulpRun* MATLAB function (<http://magnet.systemsbiology.net/tfa>).

**Computational methods**

Distributions of noise suppression (ξ and responsiveness (ρ) TFA statistics presented in Fig. 3 in the main text were calculated using a kernel density estimate. The density for each distribution was evaluated at 150 equally spaced points that cover the range of the ξ and ρ statistics. Bivariate distributions of noise suppression (ξ, ξ_1_, ξ_2_) vs. responsiveness (ρ, ρ_1_) statistics for the *OLE* and *GAL* networks presented in Fig. 2*C*-*E* and Fig. S3 were calculated using a bivariate kernel density estimator. Contour plots (Fig. 2*C*-*E* in the main text and Fig. S3) were calculated based on TFA characteristics of 3000 random stimuli/system responses (described in more detail in the main text).

**Supplementary References**

1. Ratushny AV*, et al.* (2008) Control of transcriptional variability by overlapping feed-forward regulatory motifs. *Biophysical journal* 95(8):3715-3723.

2. Ramsey SA*, et al.* (2006) Dual feedback loops in the GAL regulon suppress cellular heterogeneity in yeast. *Nat Genet* 38(9):1082-1087.

3. Litvak V*, et al.* (2009) Function of C/EBPdelta in a regulatory circuit that discriminates between transient and persistent TLR4-induced signals. *Nature immunology* 10(4):437-443.
